# The Structure of Rice Stemborer Assemblages: A Review of Species’ Distributions, Host Ranges, and Interspecific Interactions

**DOI:** 10.3390/insects14120921

**Published:** 2023-12-02

**Authors:** Finbarr G. Horgan

**Affiliations:** 1 EcoLaVerna Integral Restoration Ecology, Bridestown, Kildinan, T56 P499 Co. Cork, Ireland; f.horgan@ecolaverna.com; 2 Centre for Pesticide Suicide Prevention, University/BHF Centre for Cardiovascular Science, University of Edinburgh, Edinburgh EH16 4TJ, UK; 3 Escuela de Agronomía, Facultad de Ciencias Agrarias y Forestales, Universidad Católica del Maule, Casilla 7-D, Curicó 3430000, Chile

**Keywords:** assembly rules, conditional interspecific competition, Crambidae, Diopsidae, Diptera, grasses, Lepidoptera, Pyralidae, resource partitioning, species richness

## Abstract

**Simple Summary:**

Rice is attacked by range of stem-boring moths and flies. Despite their ecological and economic importance and the considerable scientific literature on their impacts and management, relatively little attention has been given to their coexistence mechanisms. This review lists the stemborer species associated with rice and determines their prevalence in rice crops based on published reports. The approximate distributions, host plant associations, modes of attacking rice, and details of the bionomics and behaviors of the economically important species are presented. Furthermore, using published data based on rice stem dissections, the varying structures of rice stemborer assemblages are described. Species richness is mainly determined by latitude, geography, and climate. Based on historical trends and information from stem dissections, possible mechanisms by which stemborers divide the rice crop, and thereby avoid competition, are determined. These include partitioning the resource by fidelity to rice and host plant range; by season and climate; by plant age, crop duration, and anatomy; and by the proximity and extent of alternative food resources (e.g., grasslands or other crops). Stemborer assemblages typically include a dominant primary moth species, one or more secondary species, and occasional species that are normally rare and probably spill over from adjacent grasslands. The dominance of primary species may change regionally, depending on environmental conditions including prevailing rice production systems.

**Abstract:**

This review describes global rice stemborer assemblages based on published species distributions, apparent host preferences, and reported shifts in assemblage composition in response to environmental factors. At least 56 moth (Lepidoptera: Crambidae, Pyralidae, Noctuidae) and fly (Diptera: Diopsidae, Chloropidae) species have been associated with rice; however, only 21 species are of potential, large-scale economic importance with a further 2 species of localized concern; most of the remaining species’ associations with rice are based on dubious records without economic impacts on rice production. A list of stemborer–host associations indicates that rice stemborers are largely oligophagous on grasses (Poaceae), but a few species are polyphagous (also attacking Cyperaceae, Typhaceae, and some Eudicotyledon plants). Total stemborer abundance is determined by rice cropping patterns and management. Assemblage species richness is determined by geographical location, surrounding habitat (particularly as regards secondary and occasional species), and season. Evidence suggests that stemborer assemblage structure is largely determined through conditional interspecific competition. Regional assemblages typically include a single dominant lepidopteran species (primary species) that is largely restricted to rice and for which the climate is optimal; one or more secondary species that vary based on the age of rice attacked, rice anatomy, and the proximity to other habitats (including other crops); and occasional species that probably spill over from adjacent grasslands. The co-occurrence of lepidopteran with dipteran rice stemborers requires further research attention.

## 1. Introduction

Rice is produced on about 160 million ha globally, mainly in tropical and subtropical lowlands. It is the second most important cereal crop (after maize—*Zea mays* L.) and constitutes the largest terrestrial biome in much of tropical South and Southeast Asia [1]. The rice biome also dominates much of the lowland coastal regions of tropical and subtropical Africa and the Americas, as well as the river deltas of southern Europe [2,3]. Although this biome is dominated by domesticated rice (mainly *Oryza sativa* L., but also *Oryza glaberrima* Steud. in parts of Africa), a wide range of other cereals and grasses also occur in rice production landscapes [4,5]. This considerable resource (grasses and cereal crops) hosts a diversity of arthropods that are overwhelmingly beneficial to rice ecosystems as regulators of herbivore populations and of weed biomass, as pollinators of crops and wildflowers, or as food for livestock and wildlife [5,6]. However, a relatively small number of rice-associated arthropods are also considered economically challenging because they damage rice and potentially reduce yields [7].

Among the most damaging rice herbivores are the stemborers. These include the larvae of a number of moths and flies that attack rice by boring into the plant stem where they feed and develop [7,8,9]. Stemborers can damage or kill rice tillers, giving rise to characteristic, straw-like vegetative tillers known as deadhearts, or, during the reproductive stage, result in sterile or unfilled panicles known as whiteheads [10,11,12,13]. In most rice-producing regions, stemborer assemblages consist of several species, of which a small number are associated with the greatest damage [7,9]. However, the relative economic importance of species can change over time. For example, since its introduction in the 1930s, *Chilo partellus* (Swinhoe) has become a major pest of rice and other cereals in Africa and has been associated with the declining abundance of native African stemborers [14,15]; in the 1980s, *Eoreuma loftini* (Dyar) became a major pest of rice and other crops after range expansion in Mexico and into the southern USA [16,17,18], also affecting the abundance of other stemborers [19]. Assemblages of native stemborers can also sometimes shift in species’ dominance; for example, in Japan, Korea, and China, dominance shifted from *Scirpophaga incertulas* (Walker) to *Chilo suppressalis* (Walker) beginning in the 1960s [20,21]. In contrast, in Luzon (Philippines), *S. incertulas* increased in abundance after the 1970s while the abundance of *C. suppressalis* decreased [22]. In some parts of Malaysia, dominance shifted from *Chilo polychrysus* (Meyrick) to *S. incertulas* during the 1970s [23,24], and in parts of the Philippines from *S. incertulas* to *Scirpophaga innotata* (Walker) during the 1980s [22,25]. Furthermore, in North America, *Chilo plejadellus* Zincken gained abundance relative to *Diatraea saccharalis* (Fabricius) in Texas and Louisiana during the 1970s [26]; at about that same time, *C. plejadellus* abundance and damage increased on wild rice (*Zizania* spp.) in Minnesota (USA) [27].

It is still largely unknown why shifts in stemborer assemblage structure have occurred; however, some authors have implicated changing agronomic practices and the progressive adoption of new ‘types’ of rice variety (i.e., varieties that share a common phenology or common anatomical traits) over wide areas [20,28,29]. Shifting rice production practices have also been implicated in driving regional changes in relative damage to rice from established species. For example, throughout the 1980s, the abundance and damage from *Maliarpha separatella* Ragonot declined dramatically on Madagascar when farmers shifted from long-duration to short-duration rice varieties [30] and, in China, damage from both *C. suppressalis* and *S. incertulas* increased in the 1980s and 1990s, at the same time that a majority of farmers transitioned from inbred to hybrid rice varieties [31]. Some authors have implicated changing climatic conditions as determining or facilitating shifts in cereal stemborer assemblages [21,22,32], and recent evidence suggests that temperature can determine the outcome of interspecific competition between stemborer species on a common host [15]. These shifts in species abundance and dominance indicate that stemborer assemblages are structured and restructured through changes in the quality or availability of the rice resource relative to other host plant resources, and by conditional interspecific competition.

To better understand rice stemborer resource partitioning and possible coexistence mechanisms, this paper reviews current knowledge of rice stemborer assemblages. Using published records, it assesses whether assemblages exhibit any common patterns across regions in terms of species richness, and apparent assemblage structures. As an initial step, the host ranges of stemborers are assessed and, based on this appraisal, as well as on species’ apparent fidelity to rice, the stemborers are categorized as primary (often occurs as the dominant—most abundant—species in rice, and rice is the main host), secondary (usually subordinate in rice, i.e., rarely dominant; rice is often not the main host), and occasional rice herbivores (oligophagous and polyphagous species that sometimes occur in rice). The approximate distributions of the primary and secondary herbivores are then mapped and information on assemblage compositions in different regions is presented. Furthermore, together with behavioral, bionomic, and sampling data from dissected rice plants, possible resource partitioning mechanisms are identified. The review, therefore, clarifies the nature and extent of species interactions with practical implications for rice crop management and conservation biological control, and it outlines some preliminary rules of assembly for rice stemborers.

## 2. Review Methods

An initial search of the literature was conducted at the end of June 2023 using the terms ‘stemborer’ and ‘rice’ in Google Scholar. Google Scholar was used because it retrieves a wide variety of sources from the literature including peer-reviewed scientific papers, books and book chapters, national and regional reports, conference proceedings, and unpublished theses. This wide range of documents was used to map the approximate distributions of stemborer species. After the initial search, a series of further searches were conducted using the Latin names of rice stemborer species as listed by Khan et al. (1991) [9] and Pathak and Khan (1994) [7] together with the term ‘rice’. Furthermore, the taxonomy of the species was updated and any reports of new species described from rice were added to the list. Where possible, the original reports implicating each species as a rice herbivore were checked and the status of each species’ relations to rice were revised. 

All reported field observations of the main rice stemborers species were assessed for taxonomic updates and were georeferenced. The referenced locations were then plotted and approximate species distributions traced by including all referenced points and other cited distributions. In some cases, detailed species distributions were already available [9]. Because there were a relatively large number of studies as well as available distribution data from Asia and the Americas, approximate distributions were traced without reference to national or regional boundaries (for Asia—distributions were overlaid on approximate rice distribution maps); however, many of the distribution records for African stemborers only list the species as present in specific countries; therefore, the mapped distributions for Africa were mainly based on country records for each species. 

The literature on the potential hosts of the main rice stemborers was screened for host species records. The taxonomic information related to each species was updated when required. Information from the retrieved literature was used to assess the nature of interactions (mode of attack) between the stemborers and host plants, particularly rice, to determine possible coexistence mechanisms in rice fields. At the same time, information on species biometrics and behaviors were noted to determine further, possible niche- partitioning mechanisms. Because comparative biometric data were often incomplete across stemborer species, missing data on female minimum pupal weights and wingspans were estimated using linear regressions between female pupal length, pupal weight, and wingspan for those species for which complete data were available.

To limit the review text, much of the retrieved information is presented as a series of comprehensive Supplementary Tables. It is hoped that these will serve as a resource for further research into rice stemborer management. Because of the already extensive literature on stemborers—parts of which may have been overlooked during this review, and intense, recent research interest in these species [13], the tables should be continually revised and updated. Much of the information that appears in the Supplementary Tables is summarized in the main body of the review.

## 3. Results and Discussion

### 3.1. The Economically Important Rice Stemborers

Khan et al. (1991) [9] and Pathak and Khan (1994) [7] listed a total of 46 species of lepidopteran stemborers and 5 species of dipteran stemborers that attack rice globally. However, many of the listed species are only weakly associated with rice in the source documents because reports were often based on light-trapping or sweep-netting in rice habitats and not from the rearing of larvae collected from rice samples [9,33]. As such, many of these species might be more closely associated with wild grasses or other weeds that occur in the rice fields [34,35], or with other cereal crops [34], than they are with rice. Furthermore, the taxonomy of several of the listed species has since been revised—with species often split into two or more taxa [34,35,36,37], and none of the new taxa have yet been directly associated with rice. In the case of Diopsidae flies (henceforth diopsids), a number of species from the lists of Khan et al. (1991) [9] and Pathak and Khan (1994) [7] have been amalgamated; however, diopsid taxonomy remains understudied and there is still confusion as regards the key species attacking rice and their relative distributions [9,38,39]. A small number of species can now also be added to the original lists of Khan et al. (1991) [9] and Pathak and Khan (1994) [7]. These include moth and fly species that have since been reared from rice or have emerged as rice pests in localized areas, as well as a small number of reported species that were overlooked on the lists. 

Based on the above criteria, Table 1 presents a revised list of 21 stemborers (with information on *Sesamia nonagrioides* (Lefèbvre) divided by subspecies) that are clearly associated with economic damage to rice plants over relatively large areas. A further 35 species with recent clarifications of status or with recent updates are included in Table S1. Of the 56 species listed in Table S1, there is a lack of clear evidence for rice herbivory among at least 24 of the species. Further information on rice stemborer assemblages, including species of localized interest, is presented in Section 3.3.

### 3.2. Stemborer Host Ranges

Several authors have listed the plants associated with stemborers (see Tables S2 and S3). However, there has been no systematic evaluation of host ranges across stemborer species and some of the more substantial host species lists have been derived from disparate studies, using a range of methods (e.g., oviposition experiments, rearing bioassays, field sampling, seed box tests, etc.). Further research on comparative host ranges is warranted. Based on the accumulated evidence presented in Table S2 and summarized in Table 2, all primary and secondary rice stemborers are at least oligophagous on grasses (Poaceae). A number of species have also been reported from monocotyledonous sedges and reeds (Table 2 and Table S2). Many stemborer species have been associated with one or more potential trap plants (Table S2). Trap plants are species that are used by stemborers for oviposition, but where larvae fail to develop to adults [140,141,142,143,144]. Some trap plants may be more attractive to stemborers than rice [145].

The more extensive host species lists have been compiled for the invasive species *C. partellus*, *C. suppressalis*, *D. saccharalis*, and *E. loftini*; and the major pest species *S. incertulas*, *S. nonagrioides*, and *S. inferens* (Table 2 and Table S2). However, these species have also received the greatest research attention (Table S3). The species *D. saccharalis*, *E. lignosellus*, *S. incertulas*, *S. nonagrioides*, *S. calamistis,* and *S. inferens* are associated with a diversity of hosts including a range of taxonomically distinct crops, sedges, and reeds, indicating that these species are somewhat polyphagous. A number of the secondary rice pest species from Table 1 are mainly associated with other crops including maize (e.g., *S. nonagrioides*, *C. partellus*, *S. inferens*), sugarcane (e.g., *C. auricilius*, *S. nonagrioides botanephaga*), sorghum (e.g., *C. partellus*), or with wild grasses (e.g., *C. calamistis*, *C. polychrysus*) (Table 1 and Table S1). Despite their ability to feed on other plants, the abundance of many of the primary rice stemborers is largely determined by the availability of rice [22,27,40,191].

### 3.3. Stemborer Distribution Ranges and Assemblage Structures

Species distributions and assemblage structures based on stem dissections are discussed by region in the following sections with distribution maps provided for the 21 species from Table 1.

#### 3.3.1. Africa and the Mediterranean

**Figure 1:**
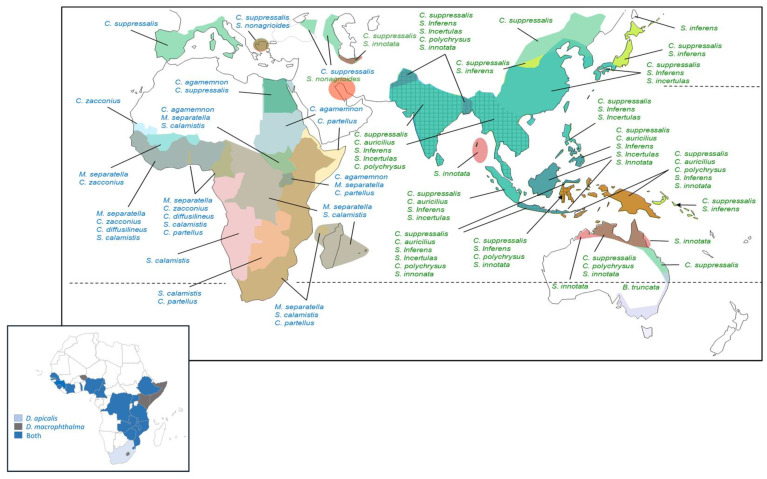
Approximate distributions of economically important rice stemborer moths in Africa and the Mediterranean (blue font), and in Asia and Oceania (green font). Shaded polygons indicate distinct stemborer assemblages occurring in rice. Distribution ranges were estimated using published field data and regional/national records as indicated with the Supplementary Information. The known distributions of the economically important rice diopsids based on information in Khan et al. (1991) [9] are indicated in the inset figure.

A total of 29 stemborer species have been associated with rice in Africa; however, there are no recent records from rice for 16 of these species (Table S1); this includes *Busseola fusca* Fuller, a major pest of maize that was likely misidentified from rice in the original source information [34,192], and *Eldana saccharina* Walker, for which a direct association with rice appears doubtful [100]. A further four species (*Ancylolomia chrysographellus* Kollar and Redtenbacher, *Adelpherupa costipunctalis* Maes, *Adelpherupa flavescens* Hampson, and *Chilo aleniellus* Strand) have been reared from rice or associated with damage to rice, but without further, recent reports [9,35,139]. Of the remaining species, some, although widespread, damage rice in relatively localized regions: For example, *S. nonagrioides* that occurs around the Adriatic Sea and Persian Gulf, although it attacks rice in southern Europe, is more commonly associated with maize [40,52,193]. The species has also been reported from sugarcane in Iran apparently without attacking adjacent rice fields [194,195]. In West Africa, the subspecies *Sesamia nonagrioides botanephaga* Tams and Bowden is also more prevalent in maize and sugarcane [32,101,166]. Similarly, the introduced species *C. partellus* is a major pest of rice in some parts of Africa [38,48]; however, it is mainly a pest of maize and sorghum in its native range in Asia [196,197], although it occasionally damages Asian rice [118]. Figure 1 indicates the approximate distributions of the nine economically important rice stemborers in Africa, as well as the European and Central Asian distributions of invasive or naturalized *C. suppressalis* [198,199]. Based on distribution records, rice stemborer assemblages in tropical West Africa may include four or five lepidopteran stemborers and one or both of the main diopsid stemborers. In East Africa, assemblages can include four or five of the main species, including lepidopterans and diopsids (Figure 1). 

**Figure 2:**
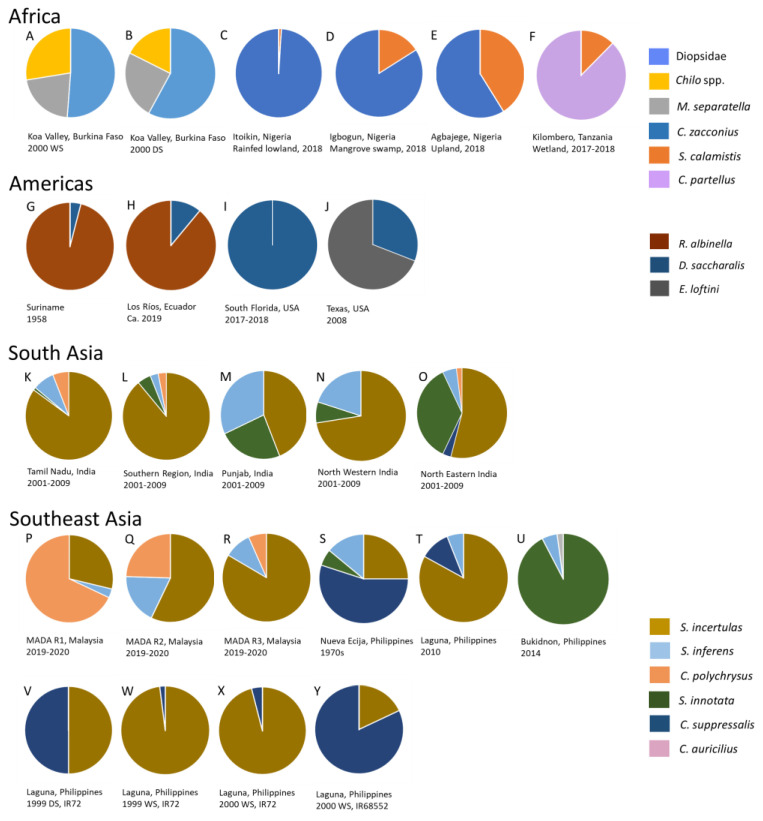
Examples of stemborer assemblage composition based on dissected rice stems from farmers’ fields and experimental field plots in Africa (**A**–**F**), the Americas (**G**–**J**), South Asia (**K**–**O**). and Southeast Asia (**P**–**Y**). Pie charts represent the proportional occurrence of each stemborer species based on numbers of individuals and are not indicative of related damage. The pie charts are based on data presented by Ba et al. (2008) [96] (**A**,**B**); Adewoye et al. (2021) [49] (**C**–**E**); January et al. (2021) [48] (**F**); Van Dither (1971) [91] (**G**); Vera Piguabe (2019) [202] (**H**); Roldan et al. (2020) [82] (**I**); Beuzelin et al. (2012) [169] (**J**); Katti et al. (2011) [67] (**K**–**O**); Khari and Hamid (2022) ([23]) (**P**–**R**); Das et al. (1976) [203] (**S**); Horgan et al. (2021) [73] (**T**); Horgan (unpublished data) (**U**); and Zhu et al. (2002) [204] (**V**–**Y**). Stemborer groups or species are indicated in the legends. DS = dry season, WS = wet season.

Diopsids are prevalent stemborers in West African rice fields [56,58,59,96] and were the most abundant species in dissected rice stems in a study from Burkina Faso [96] (Figure 2A,B), but not in a study from Cameroon [200]. Among the lepidopteran stemborers, *M. separatella* and *C. zacconius* are often the most abundant species in lowland, rain-fed rice, with *S. calamistis* or *S. diffusilineus* largely occurring as secondary species [46,54,96,201] (Figure 2C–E). Meanwhile, in East Africa, *M. separatella* is often the dominant species [30,47,98]; but *C. partellus* is dominant at sites in Tanzania, with *M. separatella, C. zacconius,* or *C. calamistis* as secondary species [48,98] (Figure 2F). Diopsids appear less prevalent from East African studies, although they occur widely throughout the region [38] (Figure 1 and Figure 2).

The relative abundance of African stemborers is affected by season (Figure 2A,B) and habitat (Figure 2C–E), without affecting dominance (i.e., the rank abundance of species is maintained, particularly for the dominant species). January et al. (2021) [48] have shown that the abundance of *S. calamistis* relative to *C. partellus* increases with soil nitrogen levels and seeding density, and Leonard et al. (2015) [98] indicate varietal and sowing-date effects on the relative abundance of *C. partellus*, *M. separatella,* and *S. calamistis* in Tanzania—in both studies, this did not affect the dominance of *C. partellus*. Although not statistically significant, rice varieties and insecticide treatments also affected the relative abundance of lepidopteran stemborers in field plots in Nigeria, without affecting dominance [54].

#### 3.3.2. Asia and Oceania

A total of 18 stemborer species have been associated with rice in Asia and Oceania (Table S1). *Scirpophaga gilviberbis* Zeller and *A. chrysographellus,* which have been associated with rice in Africa, also occur in Asia [33]. Of these 20 species, there are no direct records, or no recent records for 9 of the species feeding on rice. This includes a single report, from the 1950s, of *Niphadoses palleucus* Common attacking rice at a site in north Western Australia [122] and a single report of *Saluria inficita* (Walker) from rice in the Philippines [22]. *Scirpophaga nivella* (Fabricus) Lewvanich likely invaded New Caledonia and Fiji with the spread of rice [33]; however, there have been no recent reports of the species attacking rice in the Pacific region or in its native range in mainland Asia [9]. Seven species occur as primary or secondary species in rice (Table 1). Two further species are of localized economic importance (Table S1): *Scirpophaga fusciflua* Hampson occurs as a minor pest of rice in south India; however, in recent years it has been reported as the dominant species in rice in Himanchal Pradesh [123,128,205]. The Chloropidae fly *Anatrichus erinaceus* Loew has recently been associated with rice in Uttar Pradesh (India), where it caused up to 30% damage to rice in some localities [116]. 

Figure 1 indicates that tropical rice fields in Asia could include between three and six of the main stemborer species (see also Figure S1); however, dissections of rice stems indicate no more than five species in a given region: *S. incertulas*, *C. suppressalis,* and *S. innotata* are frequently the dominant species in Asian rice stemborer assemblages (Figure 2K–Y) [75,206]. *Chilo polychrysus* has also been reported as dominant at locations in Malaysia, particularly during older surveys (i.e., 1970s) [23,24,75,119,207] (Figure 2P). Stem dissections indicate that dominance is strongly influenced by habitat and region (Figure 2K–U), including altitude [208]. Furthermore, Zhu et al. (2002) [204] and Horgan et al. (2021) [73] indicate that this dominance can shift depending on season (Figure 2V,W) (see also Litsinger et al. (2011) [22]) and rice variety (Figure 2X,Y). Stem dissections suggest that assemblage composition is largely unaffected by crop establishment practices (drum-seeded, direct-seeded, transplanted, system of rice intensification—SRI [209]) and fertilizer inputs [72,73]. Furthermore, closely related hybrid, inbred, and sterile rice lines had little effect on stemborer assemblage structure in field plots in the Philippines [210]. The prevalence of *Sesamia inferens* (Walker) in dissected rice stems from north western India and the Punjab suggests that the abundance of this species is affected by cropping patterns including rice–wheat systems (Figure 2M,N) [29].

#### 3.3.3. North America, South America, and the Caribbean

**Figure 3:**
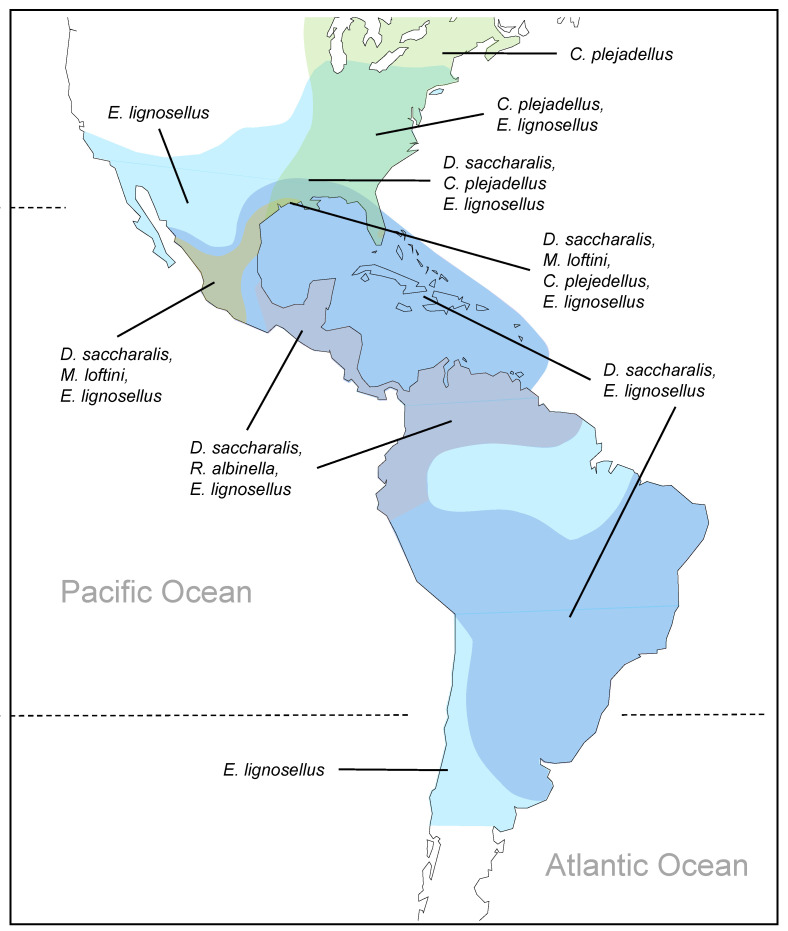
Approximate distribution of lepidopteran stemborers that attack rice and other crops in the Americas. Colored polygons indicate distinct rice stemborer assemblages.

Relatively few stemborer species damage rice in the Americas, where only six species have been reported (Table S1). Of these, only *Diatraea lineolata* (Walker) has no recent records from rice (Table S1). This species mainly occurs in maize and sugarcane [19,129]. In the Americas, the maximum diversity of rice stemborers occurs in the southern USA, Mexico, Central America, and the northern parts of South America (three–four co-occurring species) (Figure 3). *Elasmopalpus lignosellus* (Zeller) is the most widespread species, occurring from the Great Lakes to central Chile (Figure 3). The species is highly polyphagous (Table 2 and Table S2) and damages upland rice seedlings in northern Brazil [93]. *Chilo plejadellus* is restricted to the eastern USA and south eastern Canada. Around the Great Lakes, it damages wild rice (*Zizania* spp.) [27]. Although *C. plejadellus* occurs in low numbers, it was often the predominant species in Louisiana rice fields during the 1970s [26,79] when it increased in abundance relative to *D. saccharalis*, possibly in response to the adoption of relatively thick-stemmed rice varieties [78,79]. *Diatraea saccharalis* is widespread in the tropical and subtropical Americas, extending from Florida and the Gulf of Mexico (Texas, Louisiana) to southern Brazil and Uruguay. Two species with a more restricted distribution are *Rupela albinella* (Cramer), which occurs in Central America and tropical South America (Figure 3) as the dominant species in rice (Figure 2G,H), and *E. loftini,* which occurs mainly in Mexico, but has recently extended its range into the southern USA [16,17,19,211,212]. *Eoreuma loftini* is now the dominant species in Louisiana and Texas rice fields [86,87], largely displacing *D. lineolata* and *D. saccharalis* in rice and other crops in its expanded range [19] (Figure 2I,J).

### 3.4. Stemborer Abundance and Species Richness

Notable shifts in the relative abundance of rice stemborer species over time suggest that their assemblages are ultimately structured through interspecific competition. Large scale changes in agriculture (e.g., crop diversity and distribution, crop rotations, pesticide and fertilizer inputs, irrigation regimes, land clearing) [48,213,214,215,216,217,218,219,220] and rice cropping practices (e.g., extent of rice production, production intensity, crop duration, synchronization of cropping) affect resource availability, setting the carrying capacity of the environment for stemborers and influencing total stemborer abundance and consequent damage [22,27,30,31,33,40,191,221,222]. Some of these changes apparently favor some species more than others (see Section 3.5).

Regionally, the potential species richness of rice stemborer assemblages is determined by latitude, altitude, and associated habitats, and, for islands, by the proximity to a continental mainland (Figure 1, Figure 3 and Figure S1) [32,62,67,223,224]. In-field stemborer richness will be strongly influenced by the occurrence, at low densities, of occasional stem-boring species, many of which are oligophagous grass-feeders—including species that predominantly occur in other cereal crops, or polyphagous species that often occur in upland rice or in relatively dry rice production environments (Table 1 and Table S1). For example, when occasional species are included, rice in some parts of tropical Asia could be affected by up to nine stemborer species (Figure S1). Many of the occasional species have no negative economic impacts on rice production; however, some may have beneficial functions by reducing weed biomass or as alternative hosts of the important natural enemies in rice production systems [182,223,225,226]. It is also possible that some of these occasional rice-feeding stemborers could become significant rice pests in specific rice production systems or if introduced to new regions, as likely occurred with *C. partellus* in Africa or *S. fusciflua* and *A. erinaceus* in India [14,116,123,128,150]. However, the apparent rarity of occasional species, particularly in recent African studies, might also point to a general decline in biodiversity associated with cereal crop expansion and grassland clearing.

Stemborer species richness in rice is also strongly affected by the spread of crop-associated invasive species including *C. partellus*, *C. suppressalis*, *S. innotata*, *S. nivella*, *D. saccharalis*, and *E. loftini* [18,33,64,185,191,198,199,212,227,228,229,230]. Many of these species caused shifts in assemblage structures in parts of their expanded ranges, often becoming the dominant species in rice (e.g., Figure 2F,I,J). This may be due to a release from natural enemies or to other competitive advantages over native rice stemborer species.

### 3.5. Resource Partitioning and Potential Coexistence Mechanisms

Shifts in species dominance over time and in response to large-scale changes to rice production practices reveal possible assemblage structuring mechanisms [14,19,20,22]. Stem dissections from field experiments, although relatively uncommon, offer further evidence of resource partitioning (Figure 2), particularly where these can be linked to comparative reports on stemborer bionomics (Table S4) and behaviors (vis-à-vis modes of attack: Table 1). However, stemborer assemblages are often characterized by the overwhelming dominance of a single species capable of ‘displacing’ potential competitors [14,19,22]. Despite such dominance, apparently weaker competitors, with very similar modes of attack, still occur at low or moderate densities in the rice crop (Figure 2), sometimes even in the same rice plants [22,73]. This suggests that interspecific competition is seldom sufficient to exclude competitors—despite all stemborer species occurring inside the stem lumen. This paradox may be explained by a high level of aggression, intraguild predation, and cannibalism [71,73,178] that reduce intraspecific competition and limit interspecific encounters inside the rice plant [231]; and by the intraspecific aggregation of egg masses and larvae in the rice crop [134,232,233]

**Figure 4:**
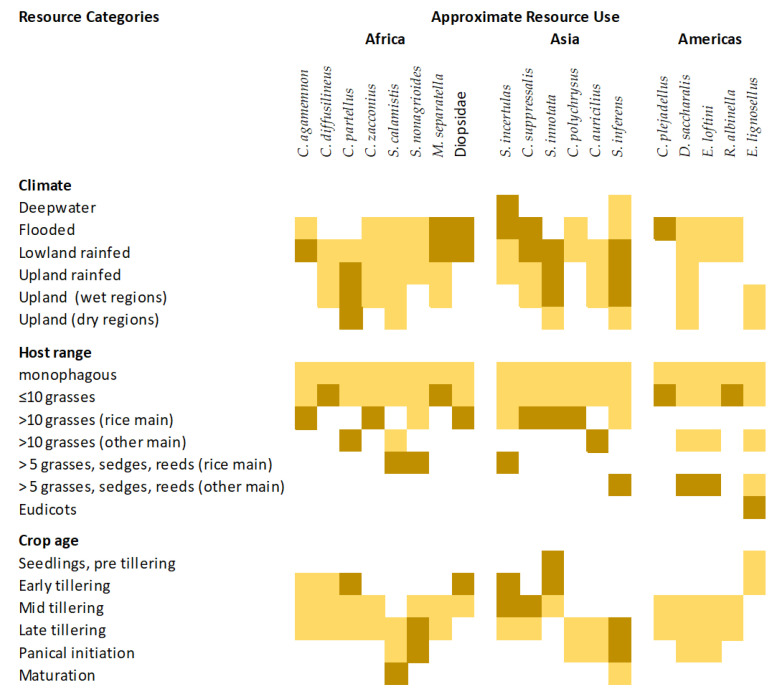
Approximate use of three resources by stemborers based on published information (darker shading indicates peak occupancy). ‘Rice main’ indicates that rice is the main host; ‘other main’ indicates that other crops are the main hosts.

Oligophagy plays a large role in stemborer species’ coexistence (Figure 4). Crop plants offer a highly favorable resource for most herbivores because of their high nutrient content (due to fertilizers), their relatively consistent access to water, and often low anti-herbivore defenses [72,73]. Oligophagy allows the survival of a number of stemborer species during periods when rice is unavailable [41,66,234]. It may also allow weaker competitors to maintain populations in the rice landscape during periods when some superior competitor is most abundant [235]. For example, the recent invasions of Fall Armyworm, *Spodoptera frugiperda* (J. E. Smith) in Africa and Asia, have been associated with a declining abundance of native stemborers in maize [236,237,238,239,240,241], even though *S. frugiperda* is not a stemborer, but a leaf chewer. This is partly due to predation of neonate stemborers before they enter the stem by *S. frugiperda* at high post-introduction densities—as shown in laboratory experiments [238,240,241]. Furthermore, *S. frugiperda* larvae have been show to induce maize defenses that subsequently reduce stemborer fitness (i.e., plant-mediated interspecific competition [238]). These interactions are predicted to shift maize stemborers from maize to relatively enemy-free or competitor-free crops such as sorghum [237,239]. Proximity to weedy grasslands or alternative crop hosts have also been suggested to influence the relative abundance of stemborers in rice, including determining the dominance of species in the assemblage [23,56,58,98,179]. However, this hypothesis is difficult to test. Although many of the primary rice stemborers are oligophagous, they occur predominantly in rice and their abundance is determined by the presence and extent of rice production [22,40,191]. Such primary pests are likely the best adapted to rice and, therefore, the strongest competitors for the rice resource over a wide range of environments. Therefore, whereas oligophagy maintains secondary and occasional stemborers in the rice environment, other partitioning mechanisms likely play a greater role in the co-occurrence of the primary species.

Evidence from Asia indicates that shifts in the dominance of the primary rice stemborers depend on production systems (e.g., the availability of standing water in the system) and climate (Figure 4). For example, seasonal and regional shifts in the dominance of *S. incertulas*, *C. suppressalis,* and *S. innotata* in the Philippines [25,204] suggest increasing advantages for the latter two species because of relatively dryer environments. Indeed, *S. innotata* can undergo prolonged diapause during drought conditions [25]. Litsinger et al. (2011) [22] suggest that *S. innotata* largely displaced *S. incertulas* and *C. suppressalis* in the southern Philippines after a particularly dry El Niño period. Similarly, *C. polychrysus* and *S. inferens* occur in deepwater systems during dryer periods of production and are displaced by *S. incertulas* when the systems are flooded [29,69,71]. In Africa, diopsid flies and *S. calamistis* are often abundant in wet, lowland environments [38,56,58,59,60]. Regional differences in weather and climate have also been implicated in the coexistence of *S. nonagrioides botanephaga* and *S. calamistis* in Côte d’Ivoire, with the former species occurring predominantly in wet coastal zones and the latter in upland savannas [32]. Similarly, *C. diffusilineus* occurs more in lowland rice fields with *C. zacconius* in the highlands [242]. 

The influence of weather and climate on the distribution and abundance of a number of rice stemborers has been studied using climate models and species’ temperature profiles [21,243,244,245]. Based on >50 years of historical weather data from southern China, Shi et al. (2012) [21] suggest that a declining abundance of *S. incertulas* could be attributed to increasing global temperatures. This hypothesis is consistent with the affinity of *S. incertulas* for wetter environments; however, the study did not include the possibility for displacement of the species by other stemborers, for which increasing temperatures might represent an advantage. For example, in experiments that manipulated stemborers under a range of temperature regimes, *S. calamistis* and *B. fusca* performed relatively poorly in the presence of *C. partellus* on maize plants and in ‘artificial stems’ under relatively high temperatures (25–30 °C) compared to lower temperatures [15,246], thereby, further indicating how interspecific competition conditioned by climate might determine relative abundance.

The time of maximum occurrence in the crop often differs among co-existing stemborer species (Figure 4). Maximum occurrence depends on oviposition preferences, larval dispersal behaviors, and intra- or interspecific aggression. Most species occur during the vegetative stages of crop development and some have poor fitness on older plants when the stems are tough and the carbon to nitrogen ratios increase. Among the earliest colonizers of rice fields are *S. incertulas*, *S. innotata,* and the diopsids [22,59] (Figure 4). Diopsids lay eggs individually over many days, in contrast to the lepidopterans that produce egg masses each with 10s or 100s of eggs (Table S4) [59]. Furthermore, the diopsids may kill rice leaves by feeding on meristems, without causing stem death [38] (Table 1). Therefore, it is unclear whether diopsids directly compete with lepidopteran stemborers. Leonard et al. (2015) [98] suggests that *C. partellus* can dominate rice stemborer assemblages in Tanzania because it occurs earlier in rice and has faster development. Indeed, recent experiments have shown that *C. partellus* performs relatively well in the presence of other stemborers and *S. frugiperda* because of its relatively fast growth rate and, consequently, limited exposure to competitors [236,246,247,248]. *Elasmopalpus lignosellus* is capable of damaging pre-tillering seedlings in upland rice systems [93]. Late colonizers include *S. nonagrioides*, *S. calamistis,* and *S. inferens* [38,41,214,224,249] (Figure 4). A recent study has shown that volatiles can determine the attractiveness of the host plant for ovipositing stemborers; however, in experiments with *S. calamistis* and *C. partellus*, the induced volatiles tended to increase the attractiveness of maize, thereby functioning as a plant-mediated facilitator of host attack [250]—and, therefore, likely increases interspecific aggregation. It is possible that similarly induced volatiles might also function to draw-in natural enemies, such as egg parasitoids [251,252,253]. Environmental effects on rice development can further influence stemborer assemblages; for example, upland rice has thicker stems at early vegetative stages that likely reduces its suitability for diopsids [60], and nitrogenous fertilizers prolong rice maturation and increase stem thickness, thereby favoring relatively late colonizers and larger species (see below) [72,73,254].

**Figure 5:**
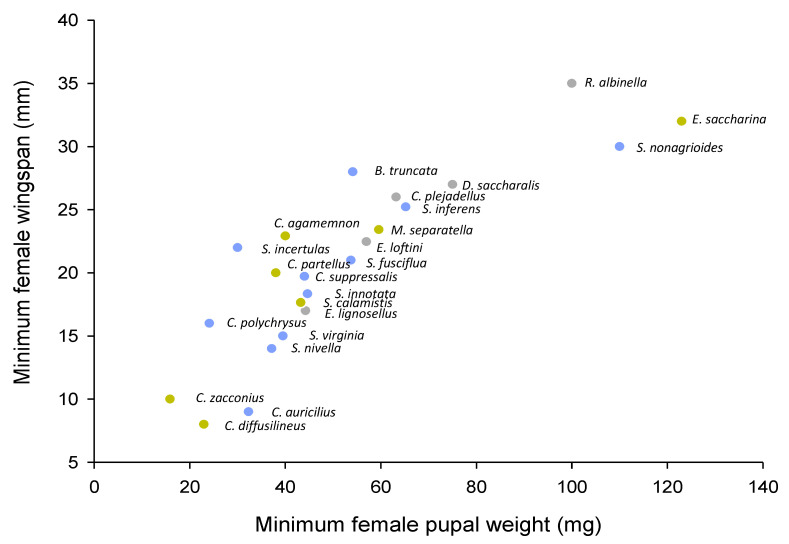
Biplot of reported minimum stemborer female pupal weights versus minimum female wingspans for African (brown symbols), Asian (blue symbols), and American (gray symbols) species. Some of the data may refer to species reared on other crops and not rice. See Table S4 for further details on species bionomics and source information [10,21,55,62,64,66,67,72,73,76,83,89,90,94,101,106,107,111,117,128,134,245,255,256,257,258,259,260,261,262,263,264,265,266,267,268,269,270,271,272,273,274,275,276,277,278,279].

Stemborers also differ in the locations they occupy in rice stems and the number of stems they occupy during infestation; for example, *C. suppressalis* occupies higher internodes than *S. incertulas* and *S. inferens*, both of which occur predominantly at the base of the stems (Table 1). Rice anatomy interacts with stemborer bionomics and life histories to determine these features of colonization. For example, the rice lumen must be sufficiently wide to accommodate stemborer development, suggesting that stemborers may be restricted by stem thickness based on the minimum size of female larvae or pupae. Because stem thickness increases with plant age, smaller stemborer species are predicted to occur earlier during crop development, or have a more rapid development than relatively larger species. Some (i.e., *S. inferens*, *S. nonagrioides*), but not all (i.e., *S. calamistis*), late colonizers are relatively large (Figure 5). Shifts in dominance between *D. saccharalis* and *C. plejadellus* in Louisiana [78,79] and between *S. incertulas* and *S. suppressalis* in Asia [22,31] have been attributed to the large-scale planting of varieties with relatively thick stems. Oliver et al. 1975 [78] found that pupae of *C. plejadellus* were often larger than *D. saccharalis;* furthermore, *S. suppressalis* are often heavier than *S. incertulas* [72,73]; however, in general, evidence to support the hypothesis for changing species’ dominance related to stem thickness is weak. Horgan et al. (2021) [73] also suggested that *C. suppressalis* and *S. incertulas* likely segregate on the basis of rice-tillering patterns, with the former more abundant on low tillering varieties, which also often have thicker stems. However, no such patterns were observed among stemborers on fertile and sterile rice, despite profuse tillering of the sterile rice [210]. This suggests that a combination of factors, including tillering, stem thickness, and others, might determine species’ preferences. 

Authors have indicated that a declining abundance of *M. separatella* in Madagascar [30], *C. polychrysus* in Malaysia [24], and *S. innotata* in Australia [22] coincided with the planting of short-duration varieties. This suggests that the species failed to complete their final generation in the rice, effectively functioning as an ecological trap. It is difficult to link crop duration with stemborer development times because systematic knowledge of rice growth and larval development times under varying temperatures is still not available (Table S4) and competition between rice herbivores across a range of temperatures can be influenced by the temperature optima of the rice varieties on which they develop [280,281]. Short duration varieties are often part of double- or triple-cropping systems, which might increase the abundance of those species with relatively short larval periods and rapid development [31]. However, this hypothesis remains untested.

## 4. Conclusions

Despite the extent of rice landscapes globally, only a few stemborer species (e.g., the moths *C. agamemnon*, *C. partellus*, *C. suppressalis*, *C. zacconius*, *D. sacharralis*, *M. separatella*, *S. incertulas*, *S. innotata,* and the fly species *D. macrophthalma*: Table 1) are of economic importance in rice production. These species can be divided into three or four principal groups, depending on whether diopsids are included as part of the same assemblage. Among lepidopteran stemborers, assemblages are structured by the presence of a single highly dominant species that is mainly adapted to feed on rice, for which the cropping system (including water availability and temperatures) is optimal and crop duration is sufficiently long to allow full larval development. Assemblages may or may not include one or more oligophagous or polyphagous secondary species that partition the resource by age, anatomy (e.g., stem thickness, tiller number), or proximity to key habitats and crops (e.g., *S. inferens* in wheat–rice systems). Finally, assemblages may include occasional oligophagous or polyphagous species that are of little economic importance to rice production and likely represent a spillover from native grasslands or other habitats. Assemblage structure is influenced by rice crop management. The abundance of stemborers is affected by rice cropping area, cropping intensity, fertilizer use, and crop synchronization, but species dominance appears resilient against changes in these production factors, although the relative abundance of species may be affected. A number of invasive species dominate stemborer assemblages in some regions. This is likely due to a release from natural enemies or other competitors after introduction. Much of our current understanding of stemborer assemblage structuring is based on empirical evidence. There is a need for further, process-oriented research to address several current hypotheses.

## Figures and Tables

**Table 1:** Stemborer species identified as primary or secondary pests of rice. For further details and a full list of possible rice-associated species, including occasional rice stemborers, see Table S1. Species of relatively localized occurrence are reported in Section 3.3.

Species	Status in Rice ^1^	Mode of Attack in Rice
Africa and the Mediterranean		
Crambidae		
*Chilo agamemnon* Bleszynski	Primary species [40,41,42]	Rice age at planting has no effect on damage [43]; some resistance as plants age [44]
*Chilo diffusilineus* (J. de Joannis)	Some early reports as primary species; mainly on upland rice [45,46], more recently appears as a secondary species	NA
*Chilo partellus* (Swinhoe)	Primary species in upland rice in some regions, secondary species in irrigated rice [10,47,48]; maize and sorghum are the main crop hosts	Early instars feed on leaf sheaths before entering the stem; late instars located in upper internodes [38]
*Chilo zacconius* Bleszynski	Primary species [49]	Mainly affects mid- to late tillering plants, some resistance as plants age [50]
Noctuidae		
*Sesamia calamistis* (Hampson)	Secondary species [49,51]	Occurs at the onset of flowering towards harvest [47]
*Sesamia nonagrioides* (Lefèbvre)	Primary species in southern Europe [52]; maize is the main crop host	Highest densities occur at about the time of head emergence [52]
*S. nonagrioides botanephaga* ^2^ Tams and Bowden	Subspecies that occurs as a secondary rice pest in West Africa [32]; sugarcane is the main crop host	NA
Pyralidae		
*Maliarpha separatella* Ragonot	Primary species [30,53,54]	Feeds on green tissues of the leaf sheath for about 5 days before entering the stem. Remains in a single stem, feeding in one or two internodes. Rarely cause whiteheads [38,55]
Diopsidae ^3^		
*Diopsis apicalis* Dalman	Secondary diopsid species [56,57]	Young plants [56,58]
*Diopsis macrophthalma* Dalman	Primary diopsid species; mainly in lowland, irrigated systems [56,59,60]	Young plants (<40 days after transplanting), feeds on meristems causing death of leaves and stems [38,58]
Asia and Oceania		
Crambidae		
*Chilo auricilius* Dudgeon	Some early reports as primary species [61]; more recently regarded as a secondary species [22,62]; shows little preference among crop hosts [63]; but is more commonly regarded as a sugarcane pest [64]	Prevalent in rice that is adjacent to other crop hosts, with the 2nd generation attacking rice [65,66]
*Chilo polychrysus* (Meyrick)	Primary species in some parts of Malaysia; mainly occurs as a secondary species [23,24,67,68]	Occurs in deepwater rice before flooding and at about harvest [69]; more prevalent at tillering in irrigated rice [70]
*Chilo suppressalis* (Walker)	Primary species [7,8,9,12,22,69,71]	Occurs at mid- to late tillering, poor development on mature plants; prevalent on thick-stemmed plants [72,73]
*Scirpophaga incertulas* (Walker)	Primary species; also prevalent in deepwater rice [7,8,9,22]	Occur at early to late tillering; prefers high-tillering plants [72,73]; eggs and pupae capable of surviving submergence [22,71]
*Scirpophaga innotata* (Walker)	Primary species; secondary species in some regions [22,62,74]	Occurs at early to late tillering [75]; capable of surviving prolonged drought through diapause [22,76]
Noctuidae		
*Bathytricha truncata* (Walker)	Primary species [77]	NA
*Sesamia inferens* (Walker)	Secondary species [22,67,73]; maize is the main crop host	Occurs at late crop stages occupying internodes near the plant base; prevalent in long-duration varieties [22,73]
North America, South America, and the Caribbean		
Crambidae		
*Chilo plejadellus* Zincken	Primary species [26,78,79]	Ealy larvae damage leaves and leaf sheaths, older larvae bore into the stems [27,79]
*Diatraea saccharalis* (Fabricius)	Occurs as the only stemborer in some parts of its range; prevalent in upland rice in some parts of northern Brazil [80], secondary species in the humid tropics [81,82,83,84]; sugarcane is the main crop host	Prevalent during stem elongation and panicle initiation; oviposition preference for older plants [84,85]
*Eoreuma loftini* (Dyar)	Primary species [86,87]; sugarcane is preferred for oviposition [88]	Prefers older vegetative plants for oviposition; larvae occur about 20 cm above the plant base [89,90]
*Rupela albinella* (Cramer)	Primary species in irrigated rice [83,91]	Prefers older vegetative plants for oviposition, will oviposit on younger plants but plants 40–60 days of age have optimal lumen space; can occur in internodes below water [83,92]
Pyralidae		
*Elasmopalpus lignosellus* (Zeller)	Primary species in upland rice [93]	Eggs are deposited below the soil surface, larvae bore into the stem and tunnel upwards [93,94]; can attack and kill seedlings before tillering [93]

^1^: Status in rice is based on reports of prominence; for details on occasional species see Table S1 and associated references [9,10,22,23,30,33,34,35,40,41,45,46,47,49,51,52,57,58,59,61,69,70,72,77,78,82,83,95,96,97,98,99,100,101,102,103,104,105,106,107,108,109,110,111,112,113,114,115,116,117,118,119,120,121,122,123,124,125,126,127,128,129,130,131,132,133,134,135,136,137,138,139]; for information on occurrence in other crops see Table S2. ^2^: *Sesamia nonagrioides botanephaga* Tams and Bowden is considered a subspecies. ^3^: Diopsidae are a family of flies (Diptera), all other species on this table are Lepidoptera.

**Table 2:** Host ranges of the 21 main rice stemborer species based on reported associations. Numbers indicate the total number of stemborer-associated plants as listed in Table S2 ^1^.

Stemborer Species	Poaceae (Grasses) ^2^	Other Monocots (e.g., Sedges and Reeds) ^2^	Eudicots ^2^	Crop Species	Total
Africa					
*C. agamemnon*	8	1		5	9
*C. diffusilineus*	8			6	8
*C. partellus*	34			8	34
*C. zacconius*	13			4	13
*D. apicalis*	5	2		3	7
*D. macrophthalma*	24	1		3	25
*M. separatella*	10			4	10
*S. calamistis*	54	3		7	57
*S. nonagrioides*	20	9		6	29
*S. nonagrioides botanephaga*	13	1		5	14
Asia and Oceania					
*B. truncata*	10			4	10
*C. auricilius*	16	1		5	17
*C. polychrysus*	30	2		7	32
*C. suppressalis*	53	4	6	15	63
*S. incertulas*	47	12	1	6	60
*S. inferens*	74	11	2	15	87
*S. innotata*	11	3		4	14
Americas					
*C. plejadellus*	6			3	6
*D. saccharalis*	75	8		8	83
*E. lignosellus*	21	2	12	20	35
*E. loftini*	31	3		8	35
*R. albinella*	3			1	3

^1^: See Tables S2 and S3 for full information on potential host plants and related references [9,10,33,37,38,41,45,46,55,56,58,63,64,65,66,67,83,88,89,90,94,95,96,100,101,107,123,128,129,140,141,143,144,145,146,147,148,149,150,151,152,153,154,155,156,157,158,159,160,161,162,163,164,165,166,167,168,169,170,171,172,173,174,175,176,177,178,179,180,181,182,183,184,185,186,187,188,189,190]; ^2^: Includes crop species.

## Data Availability

No new data were created; tabulated data from existing publications are included, together with source references, in the Supplementary Materials.

## Supplementary Materials

The following supporting information can be downloaded at: https://www.mdpi.com/article/10.3390/insects14120921/s1, Table S1: List of stemborers species associated with rice; Table S2: Stemborer–host plant associations; Table S3: Data sources for Table S2; Table S4: Bionomic data for key rice stemborers; Figure S1: Species richness of stemborer assemblages attacking rice in Asia. The green area indicates the main rice growing regions of Asia, including around the Caspian Sea. Species richness is based on distributions presented in Figure 1 and in the source information cited in Table S1.
