# Supplementary material for: The Structure of Rice Stemborer Assemblages: A Review of Species’ Distributions, Host Ranges, and Interspecific Interactions
Source: Insects. 2023 Dec 2;14(12):921. doi: 10.3390/insects14120921 (PMC10744004; doi:10.3390/insects14120921)
Supplement: Supplementary file 1 [file insects-14-00921-s001.zip › insects-2737570-supplementary.pdf]

Supplementary information

Table S1 List of stemborers species associated with rice[1]

Taxonomy	Species	Status in Rice
	Africa and Mediterranean	
Crambidae	*Adelpherupa costipunctalis* n. sp. Maes	Has been reared from rice in Malawi; likely a minor pest of rice [1]
Crambidae	*Adelpherupa flavescens* Hampson	Possibly a species reported from rice in Cameroon during the 1950s; has been reared from rice; No recent reports from rice [1]
Crambidae	*Ancylolomia chrysographellus* Kollar & Redtenbacher	Regarded as a minor pest of rice with a wide distribution [2]
Crambidae	*Chilo agamemnon* Bleszynski	Economic pest of rice in northeast Africa and the Middle East [3,4]
Crambidae	*Chilo aleniellus* (Strand)	Reported from rice in Côte d'Ivoire [5]; likely a minor rice pest
Crambidae	*Chilo diffusilineus* (J. de Joannis)	Reported from rice in East Africa [6]; regarded as a principal pest in upland rice in Senegal [7,8]; Burkina Faso [9]
Crambidae	*Chilo luniferalis* Hampson	Reported from rice [10]; No recent reports
Crambidae	*Chilo mesoplagalis* (Hampson)	Reported from rice [10]; No recent reports
Crambidae	*Chilo partellus* (Swinhoe)	Economic pest of rice in Uganda [11]; pest of upland rice in Kenya [12]; most abundant stemborer in light traps near rice in Tanzania [13]
Crambidae	*Chilo psammathis* (Hampson)	Reported from rice [10]; No recent reports
Crambidae	*Chilo zacconius* Bleszynski	Economic pest of rice; most abundant rice stemborer in many African countries [14]; Benin [15]; Burkina Faso [9] regarded as a principal pest in Senegal [4,7]; principal pest in Nigeria [16]
Crambidae	*Coniesta ignefusalis* (Hampson)	No recent reports from rice [17]
Crambidae	*Eldana saccharina* Walker	Reported as a minor pest of rice – no recent reports [17]; Some suggestions that it may not attack rice [18]
Crambidae	*Scirpophaga gilviberbis* Zeller	Report of association with rice, but few recent records – likely rare [19,20]
Crambidae	*Scirpophaga melanoclista* Meyr.	Reported as a frequent pest of rice in Cote d'Ivoire, but without recent updates [21,22]
Crambidae	*Scirpophaga occidentella* (Walker)	Specimens associated with rice [19] reported as widespread in rice fields [2]– no recent reports
Crambidae	*Scirpophaga subumbrosa* Meyrick	Reported from rice [23]; No recent records
Noctuidae	*Busseola fusca* Fuller	Possible misidentification on rice [24]
Noctuidae	*Sesamia calamistis* (Hampson)	Occurs in rice at relatively low densities in Benin [15], Nigeria [14] and Kenya, occurs at onset of flowering toward harvest [12]
Noctuidae	*Sesamia cretica* Lederer	Reported from rice [25]; No recent reports
Noctuidae	*Sesamia epunctifera* Hampson	No recent reports from rice
Noctuidae	*Sesamia nonagrioides* (Lefèbvre)[3]	Occurs on rice in the Middle East and considered an economic pest of rice in southern Europe [26,27]
Noctuidae	*Sesamia nonagrioides botanephaga* Tams & Bowden[2]	Economic pest of rice [28]
Noctuidae	*Sesamia penniseti* Tams & Bowden	No recent reports from rice
Pyralidae	*Maliarpha separatella* Ragonot[3]	Economic pest of rice in Africa; sometimes considered monophagous in rice [11,17,29,30]; rarely causes whiteheads [31]; most abundant species in rice in West Africa [32]; major pest in Nigeria [16]; Benin [15]; Burkina Faso [9]; Côte d'Ivoire [33];

		abundant in Tanzania [13]; most abundant species on rice in Kenya [12]; abundant in Madagascar [34];
Diopsidae	*Diopsis apicalis* Dalman	Economic pest of rice in Africa at relatively low densities [35]; occasional pest of rice in Guinee (mainly in transplanted rice) [36]
Diopsidae	*Diopsis circularis* Macquart[4]	Rice [2,37]; no recent records
Diopsidae	*Diopsis ichneumonea* Linneas[4]	Rice [2,37]; no recent records
Diopsidae	*Diopsis macrophthalma* Dalman[5]	Economic pest in Africa [35,38]; major pest in Nigeria [16,32,39]; constitutes 90% of Diopsids in Nigeria [32]; infests during vegetative stage in Kenya [12]
Diopsidae	*Diopsis servillei* Macquart	Rice [2,37]; no recent records
Asia and Oceania		
Chloropidae	*Anatrichus erinaceus* Loew	Emerging economic pest of rice in parts of India [40]
Crambidae	*Chilo auricilius* Dudgeon	Economic (minor) pest of rice [41]; was reported as the most abundant species in Taiwan in 1970s [42]
Crambidae	*Chilo partellus* (Swinhoe)	Minor pest of rice [43]
Crambidae	*Chilo polychrysus* (Meyrick)	Economic pest of rice/deep water rice; often the most abundant species in irrigated rice in Malaysia [44-48]
Crambidae	*Chilo sacchariphagus indicus* (Kapur)	No clear records from rice
Crambidae	*Chilo suppressalis* (Walker)	Economic pest of rice [49]
Crambidae	*Niphadoses palleucus* Common	No recent reports; occurred in minor proportions in the 1940-1950s at one site in the Northern Territories, Australia [50]
Crambidae	*Scirpophaga aurivena* (Hampson)	No clear records from rice
Crambidae	*Scirpophaga fusciflua* Hampson	Gaining prominence in rice in Kerala and Himanchal Pradesh, India in recent years [51,52]; Reported as the dominant species in Himanchal Pradesh by Tandon & Srivistava [53,54]
Crambidae	*Scirpophaga incertulas* (Walker)[6]	Economic pest of rice/deep water rice [44,55]
Crambidae	*Scirpophaga innotata* (Walker)	Economic pest of rice [56]
Crambidae	*Scirpophaga lineata* (Butler)	No reports from rice fields
Crambidae	*Scirpophaga nivella* (Fabricus) Lewvanich	Associated with rice fields [19]; but no recent reports of rearing from rice
Crambidae	*Scirpophaga virginia* Schultze	Recent record of association with rice in Tamil Nadu; but not reared from rice [57]
Noctuidae	*Bathytricha truncata* (Walker)	Economic pest of rice in New South Wales, Australia [58]
Noctuidae	*Sesamia inferens* (Walker)	Economic pest of rice/deep water rice [44,55]
Noctuidae	*Sesamia uniformis* Dudgeon	No recent records
Pyralidae	*Saluria inficita* (Walker)	Rice in dryland environments in the Philippines [59]; no further reports
Americas and the Caribbean		
Crambidae	*Chilo plejadellus* Zincken	Economic pest of rice [60]
Crambidae	*Diatraea lineolata* (Walker)	No recent reports from rice [61]
Crambidae	*Diatraea saccharalis* (Fabricius)	Occurs in rice [61,62]
Crambidae	*Eoreuma loftini* (Dyar)	Economic pest of rice [63]
Crambidae	*Rupela albinella* (Cramer)[7]	Appears largely restricted to rice [64,65]
Pyralidae	*Elasmopalpus lignosellus* (Zeller)	Economic pest of upland rice, particularly in Brazil [66,67]

1: Minor damage to rice by stemborer beetles (i.e., *Anadastus filiformis* (Fabricius)) has also been reported [68].

2: *Sesamia nonagrioides botanephaga* Tams & Bowden is regarded here as a subspecies of *Sesamia nonagrioides* (Lefèbvre) that occurs mainly in Africa and possibly around the Persian Gulf.

3: Specimens in Papua New Guinea and India were incorrectly identified and are *M. fuscicostella* Cook and *M. longisignumella* Cook, respectively [69]; Reported pest of sugarcane [70] and sorghum [71] in India; but Heinrichs and Barrion (2004)[31] suggests that the latter is probably based on misidentifications; not known from rice in Asia

4: Possibly subspecies of *Diopsis apicalis* Dalman [32]

5: *Diopsis macrophthalma* Dalman is regarded here as Synonymous with *Diasemopsis macrophthalma* [Dalman], *Diopsis longicornis* Macquart and *Diopsis thoracica* Westwood ([72])

6: *Catagela adjurella* Walker regarded here as synonymous with *Scirpophaga incertulas* (Walker) [2]

7: Sometimes interchanged with *Rupela albina* Becker & Solis

Table S2 Stemborer-host plant associations[1]

Plants	Stemborers																																			
	Ancylolomia chrysographella (Kollar)	*Bathytricha truncata* Walker	*Busseola fusca* (Fuller)	*Chilo agamemnon* (Bleszynski)	*Chilo aleniellus* (Strand)	*Chilo auricilius* (Dudgeon)	*Chilo diffusilineus* (J. de Joannis)	*Chilo partellus* (Swinhoe)	*Chilo plejadellus* (Zincken)	*Chilo polychrysus* (Meyrick)	*Chilo sacchariphagus indicus* (Kapur)	*Chilo suppressalis* (Walker)	*Chilo zacconius* Bleszynski	*Coniesta ignefusalis* (Hampson)	*Diatraea lineolata* (Walker)	*Diatraea saccharalis* (Fabricius)	*Elasmopalpus lignosellus* (Zeller)	*Eldana saccharina* (Walker)	*Eoreuma loftini* (Dyar)	*Maliarpha separatella* Ragonot	*Rupela albinella* (Cramer)	*Scirpophaga fusciflua* (Hampson)	*Scirpophaga gilviberbis* Zeller	*Scirpophaga incertulas* (Walker)	*Scirpophaga innotata* (walker)	*Scirpophaga nivella* (Fabricius)	*Scirpophaga virginia* (walker)	*Sesamia botanephaga* Tams & Bowden	*Sesamia calamistis* Hampson	*Sesamia cretica* Lederer	*Sesamia epunctifera* Hampson	*Sesamia inferens* (Walker)	*Sesamia nonagrioides* (botanephaga) (Lefebvre)	*Sesamia penniseti* Tams & Bowden	*Sesamia uniformis* Dudgeon	*Diopsis apicalis* Dalman	*Diopsis macrophthalma* Dalman
Eudicot																																					
Amaranthaceae																																					
Amaranthus sp.												1																									
Asteraceae																																					
Xanthium pensylvanicum Wallr.												1																									
Brassicaceae																																					
Brassica campestris L.												1																									
Brassica oleracea var. *capitata* L.																	1																				
Raphanus raphanistrum subsp. *sativus* (L.) Domin																	1																				
Euphorbiaceae																																					
Manihot esculenta Crantz																		1																			
Fabaceae																																					
Arachis hypogaea L.																	1																				
Cajanus cajan (L.) Hurt																	1																				
Glycine max L. (Merr.)																	1																				
Lablab purpureus (L.) Sweet																	1																				

Phaseolus linearis H.B.K.																	1																				
Phaseolus lunatus L.																	1																				
Phaseolus sp.																	1																				
Phaseolus vulgaris L.																	1																				
Pedaliaceae																																					
Sesamum indicum L.																	1																				
Polygonaceae																																					
Fagopyrum esculentum Moench																	1																				
Reynoutria japonica Houtt.												1																									
Rumex crispus L.																																1					
Rumex sp.																																1					
Solanaceae																																					
Solanum lycopersicum L.												1																									
Solanum melongena L.												1																									
Monocots																																					
Araceae																																					
Caladium sp.																										1											
Colocasia esculenta (L.) Schott												1																									
Cannaceae																																					
Canna sp.																			1																		
Cyperaceae																																					
Actinoscirpus grossus (L.f.) Goetgh. & D.A.Simpson						1				1		1				1								1								1					
Bolboschoenus maritimus (L.) Palla																																1					
Carex sp.																																	1				
Cyperus alopecuroides Rottb.				1														1																			
Cyperus amuricus Maxim.																																1					
Cyperus articulatus L.																																	1				
Cyperus compactus Retz.																								1													
Cyperus compressus L.																								1													
Cyperus difformis L.																								1													1
Cyperus digitatus Roxb.										1		1																				1					

Cyperus distans L.f.																1		1											1				1				
Cyperus dives Delile																		1															1				
Cyperus dubius Rottb.																								1													
Cyperus elatus L.																								1													
Cyperus esculentus L.																	1	1																			
Cyperus fastigiatus Rottb.																		1																			
Cyperus iria L.																								1													
Cyperus latifolius Poir.																		1																			
Cyperus luzulae (L.) Retz.																1																					
Cyperus natalensis Hochst. ex C.Krauss																		1																			
Cyperus papyrus L.																		1															1				
Cyperus polystachyos Rottb.																		1																			
Cyperus prolifer Lam.																		1																			
Cyperus rotundus L.																1	1	1							1							1	1				
Cyperus ruwenzoriensis (C.B.Clarke) Huygh																		1																			
Cyperus serotinus Rottb.																																1					
Cyperus sexangularis Nees																		1																			
Cyperus sp.																			1					1	1	1			1			1	1			1	
Cyperus textilis Thunb.																		1																			
Eleocharis dulcis (Burm.f.) Trin. ex Hensch.																									1											1	
Eleocharis geniculata (L.) Roem. & Schult.																1																					
Eleocharis sp.																										1											
Fimbristylis littoralis Gaudich.																								1													
Fuirena umbellata Rottb.																		1																			
Hypolytrum nemorum (Vahl) Spreng.																								1													
Kyllingia spp.																		1											1								
Mariscus thunbergii (Vahl) Schrad.																		1																			
Schoenoplectiella juncoides (Roxb.) Lye																								1													
Schoenoplectiella lateriflora (J.F.Gmel.) Lye																								1													

Schoenoplectus lacustris (L.) Palla																															1					
Schoenoplectus tabernaemontani (C.C.Gmel.) Palla																		1																		
Scirpus sp.																		1							1						1					
Scleria bracteata Cav.															1																					
Scleria pterota Presl															1																					
Poaceae																																				
Andropogon fastigiatus Sw.															1																					
Andropogon glomeratus (Walt.) Britton, Sterns & Poggenb.															1			1																		
Andropogon pratensis Hochst. ex Hack																														1						
Andropogon schirensis Hochst. ex A.Rich.																																1				
Andropogon sp.													1		1													1								
Andropogon tectorum Schumach. & Thonn																			1																	
Aristida stricta Michx.																1																				
Arundo donax L.																		1																		
Avena fatua L.																1																				
Avena sativa L.																1													1		1	1				
Axonopus capillaris (Lam.) Chase															1																					
Axonopus compressus (Sw.) P.Beuv.															1	1							1													
Beckeropsis uniseta (Nees) K.Schum.																												1								
Beckmannia eruciformis (L.) Host																															1					
Bothriochloa bladhii (Retz.) S.T.Blake																		1																		
Bothriochloa longipaniculata (Gould) Alfred & Gould																		1																		
Brachiaria distachya (L.) Stapf									1																											
Brachiaria mutica (Forssk.) Stapf															1								1													
Brachiaria plantaginea (Link) Hitchc.															1																					

Bromus sp.																			1																		
Calamagrotis epigejos (L.) Roth																																1					
Cenchrus americanus (L.) Morrone			1	1			1	1			1	1						1	1						1				1	1		1		1			
Cenchrus ciliaris L.								1																													
Cenchrus echinatus L.																1													1								
Cenchrus latifolius (Spreng.) Morrone																1																					
Cenchrus polystachios (L.) Morrone subsp. *polystachios*																													1								
Cenchrus purpureus (Schumack.) Morrone								1				1						1											1		1		1	1			
Cenchrus sp.																1													1								
Cenchrus trachyphyllum Pilg.												1																									
Chionacne sp.								1																													
Chloris gayana Kunth		1																																			
Chloris pilosa Schumach.																																					1
Chloris polydactyla (L.) Sw.																1																					
Chrysopogon sp.																													1								
Chrysopogon zizanioides (L.) Roberty								1		1	1	1				1								1					1			1					
Coelorachis glandulosa (Trin.) Stapf ex Ridl.										1																						1					
Coelorachis sp.																																1					
Coix aquatica Roxb.												1																									
Coix lacryma-jobi L.								1				1				1		1						1					1			1					
Coix sp.												1																	1								
Cortaderia selloana (Schult. & Schult.f.) Asch. & Graebn.																			1																		
Cymbopogon caesius subsp. *giganteus* (Chiov.) Sales																																					1
Cymbopogon citratus (DC.) Stapf																1			1																		
Cymbopogon nardus (L.) Rendle											1					1																1					
Cymbopogon schoenanthus Spreng																1																1					

Cynodon dactylon (L.) Pers.																	1		1					1	1												1
Dactyloctenium aegyptium (L.) Willd.																																					1
Dactyloctenium sp.		1																																			
Dichanthium aristatum (Poir.) C.E.Hubb.																1			1																		
Digitaria adscendens (H.B.K.) Henr.																																					1
Digitaria insularis (L.) Fedde																1	1																				
Digitaria milanjiana (Rendle) Stapf		1																													1						
Digitaria sanguinalis (L.) Scop.																	1		1					1													
Digitaria setigera Roth.																1																					
Echinochloa colona (L.) Link										1		1				1								1													1
Echinochloa colonum (L.)												1				1			1													1	1				
Echinochloa crus-galli (L.) P.Beauv.				1				1		1		1	1			1	1		1													1					
Echinochloa frumentacea Link								1																								1					
Echinochloa haploclada (Stapf) Stapf								1																					1								
Echinochloa polystachya (Kunth) Hitchc.																1																					
Echinochloa pyramidalis (Lam.) Hitchc. & Chase								1					1			1				1				1				1	1				1	1			1
Echinochloa sp.												1																	1			1				1	
Echinochloa stagnina (Retz.) P.Beauv.				1														1														1	1				1
Eleusine coracana Gaertn.			1					1		1								1										1	1			1					
Eleusine indica (L.) Gaertn.								1		1		1				1	1							1					1			1					
Eleusine sp.																1													1			1					
Eragrostis cilianensis (All.) Vign. ex Janchen																																1					
Eragrostis sp.																																1					
Eragrostis unioloides (Retz.) Nees ex Steud.																1								1													
Erianthus arundinaceus (Retz.) Jesw.																																1			1		
Erianthus sp.																																1					

Eriochloa meyeriana (Nees) Pilg.																																	1				
Eriochloa procera (Retz.) C.E.Hubb						1				1		1				1								1								1					
Eriochloa villosa (Thum.) Kunth																																1					
Gynerium sagittatum (Aubl.) Beauv.																1																					
Hackelochloa granularis (L.) Kuntze																1																					
Hemarthria compressa (L. f.) R.Br.						1		1																				1	1			1					
Hordeum vulgare L.																	1		1											1		1					
Hymenachne amplexicaulis (Rudge) Nees.										1						1																					
Hymenachne donacifolia (Raddi) Chase																1																					
Hymenachne sp.																1																1					
Hyparrhenia sp.																													1								
Hyparrhenia rufa (Nees) Stapf			1					1								1													1								
Hyparrhenia tamba (Steud.) Stapf								1																													
Imperata cylindrica (L.) Beauv	1																								1												1
Ischaemum aristatum L.								1																1													
Ischaemum rugosum Salisb.												1													1							1					
Ischaemum sp.																																1					
Ischaemum timorense Kunth										1		1												1								1					
Leersia hexandra Sw.																								1													1
Leptochloa chinensis (L.) Nees						1																		1													
Leptochloa mucronata (Michx.) Kunth																1																					
Leptochloa nealleyi Vasey																			1																		
Leptochloa panicea (Retz.) Ohwi																1																					
Leptochloa panicoides (J.Presl) Hitchc.																1			1					1													
Leptochloa scabra Nees																1																					
Leptochloa virgata (L.) Beauv.																1																					

Lepturus repens (Forst.F.) R.Br.			1					1										1											1								
Lepturus sp.																													1								
Lolium sp.																			1										1								
Luziola fluitans (Michx.) Terrell & H.Rob.																	1																				
Luziola spruceana Benth. ex Döll																1																					
Megathyrsus maximus (Jacq.) B.K.Simon & S.W.L.Jacobs		1						1					1																1		1	1					
Melinis repens (Willd.) Zizka		1														1																					
Miscanthus fuscus (Roxb.) Benth.						1					1	1																				1			1		
Miscanthus sacchariflorus (Maxim.) Franch.												1																									
Miscanthus sinensis Andersson												1													1							1					
Miscanthus sp.																																1					
Oryza australiensis Domin																								1	1												
Oryza barthii A.Chev.												1	1							1				1												1	1
Oryza eichingeri Peter												1												1													
Oryza glabberima Steud.													1							1				1													1
Oryza grandiglumis (Döll) Prodoehl																								1													
Oryza granulata Nees & Arn. ex Watt																								1													
Oryza latifolia Desv.						1				1		1				1								1								1					
Oryza longistaminata A.Chev. & Roehr.							1						1							1				1									1				1
Oryza minuta J.C.Presl ex C.B.Presl										1		1												1								1					
Oryza nivara S.D.Sharma & Shastry												1								1				1													
Oryza oficinalis Wall. ex Watt												1												1													
Oryza perennis Moench												1				1								1													
Oryza punctata Kotschy ex Steud.												1								1				1													
Oryza ridleyi Hook.f.												1																									
Oryza rufipogon Griff.												1												1	1												

Oryza sativa L.	1	1	1	1	1	1	1	1	1	1	1	1	1	1	1	1	1		1	1	1	1	1	1	1	1	1	1	1	1	1	1	1	1	1	1	1
Panicum amplexicaule Rudge										1																						1					
Panicum auritum J. Presl ex Nees										1		1												1								1					
Panicum dichotomiflorum Michx.																1																					
Panicum elephantipes Nees ex Trin.																1																					
Panicum grande Hitchc. ex Chase																1																					
Panicum gymnocarpum Elliott																1																					
Panicum maximum Jacq.			1								1		1			1	1												1			1		1			
Panicum merkeri Mez												1																			1	1					
Panicum miliaceum L.												1							1													1					
Panicum repens L.										1		1												1								1					1
Panicum spp.							1										1												1			1					
Panicum trichocladum Hack. ex K. Schum.																																	1				
Paspalidium geminatum (Forssk.) Stapf																1																					
Paspalidium punctatum (Burm.f.) A.Camus										1														1								1					
Paspalum conjugatum P.J.Bergius																													1								
Paspalum densum Poir.																1																					
Paspalum denticulatum Trin.																			1																		
Paspalum dichotomiflorum Michx.																			1																		
Paspalum dilatatum Poir.	1	1																	1																		
Paspalum distichum L.												1																									
Paspalum fasciculatum Willd. ex Flüggé																1																					
Paspalum millegranum Schrad.																1																					
Paspalum orbiculare G.Forst.																																1					
Paspalum plicatulum Michx.																1																					
Paspalum punctatum Flüggé												1																				1					
Paspalum repens P.J.Bergius																1																					

Paspalum scrobiculatum L.							1			1		1												1								1					1
Paspalum secans Hitchc. & A.Chase																1																					
Paspalum sp.														1		1													1			1				1	
Paspalum thunbergii Kunth ex Steud.																																1					
Paspalum uruillei Steud.																1		1	1										1								
Paspalum virgatum L.																1																					
Pennisetum pedicellatum Trin.																																					1
Pennisetum polystachion (L.) Schult.																													1				1				
Pennisetum purpureum Schumach.			1					1		1						1		1										1	1					1			
Pennisetum sp.													1	1			1												1			1					
Pennisetum thunbergii Kunth																																	1				
Phacelurus gabonensis Steud. Clayton																																					1
Phalaris arundinacea L.																													1								
Phalaris sp.																			1										1								
Phragmites australis (Cav.) Trin. ex Steud.				1								1																					1				
Phragmites karka (Retz.) TM. ex Steud.												1																				1					
Phragmites sp.																																1					
Pleioblastus simonii (Carr.) Nakai												1																									
Polypogon fugax Nees ex Steud.																																1					
Polypogon sp.																																1					
Polytrias indica (Houtt.) Veldkamp																								1													
Pseudechinolaena polystachya (Kunth) Staph.																1																					
Rottboellia cochinchinensis (Lour.) Clayton													1					1										1	1					1			1
Rottboellia compressa L.f.								1																					1				1				
Rottboellia exaltata L.f.																		1																			1
Rottboellia sp.																													1								
Saccharum officinarum L.		1	1	1		1	1	1		1	1	1	1		1	1	1	1	1	1				1	1	1		1	1	1		1	1		1		

Saccharum spontaneum L.						1					1													1					1		1			1		
Saccharum sp.										1		1						1										1			1					
Sacciolepis myosuroides (R.Br) A.Camus						1				1		1												1							1					
Sacciolepis myuros (Lam.) Chase						1				1														1							1					
Sacciolepis sp.										1						1															1					
Setaria barbata (Lam.) Kunth																1								1				1								1
Setaria italica (L.) P. Beauvois										1																					1					
Setaria megaphylla (Steud.) T.Dur. & Schinz																												1					1			
Setaria parviflora (Poiret) Kerguélen										1		1																								
Setaria pumila (Poir.) Roem. & Schult.						1				1		1				1			1					1							1					
Setaria sphacelata (Schumach.) Stapf & C.E.Hubb. ex M.B.Moss																												1					1			1
Setaria sp.																											1	1			1					
Setaria verticillata (L.) P.Beauv.																																				1
Sorghastrum stipoides (Kunth) Nash																											1									
Sorghum arundinaceum (Desv.) Stapf			1					1					1					1									1	1		1						
Sorghum bicolor (L.) Moench			1	1		1	1	1				1		1		1	1	1	1	1							1	1	1		1	1	1	1	1	
Sorghum halepense (L.) Pers.						1		1				1				1	1		1									1	1		1	1				
Sorghum sp.																												1								
Sorghum × drummondii (Nees ex. Steud.) Millsp. & Chase								1				1				1	1														1					
Spartina alterniflorus (Loisel) P.M.Peterson & Saarela									1																											
Sporobolus cynosuroides (L.) P.M.Peterson & Saarela									1																											
Sporobolus marginatus Hochst. ex A.Rich.								1																												
Themeda triandra Forssk.																								1												

Tripidium arundinaceum (Retz.) Welker, Voronts. & E.A.Kallogg						1		1				1													1							1			1		
Tripidium bengalense (Retz.) H.Scholz								1																													
Tripidium sp.																1																					
Tripsacum dactyloides (L.) L.																1																					
Tripsacum laxum Nash															1	1													1								
Tripsacum sp.																													1								
Tristachya leucothrix Nees																								1													
Triticum aestivum L.		1								1		1				1	1							1					1			1					
Triticum durum Desf.																																	1				
Triticum sp.															1									1					1	1		1			1		
Uniola paniculata Roth																1																					
Urochloa brizantha (Hochst. ex A.Rich.) R.Webster								1																													
Urochloa fusca B.F.Hansen & Wunderlin																1			1																		
Urochloa lata (Schumach.) C.E.Hubb																																					1
Urochloa platyphila (Munro ex C.Wright) R.D.Webster																			1																		
Urochloa subquadripara (Trin.) R.D.Webster																								1													
Vossia cuspidata (Roxb.) Griff.			1					1																				1	1				1				
Zea mays L.		1	1	1	1	1	1	1		1	1	1	1	1	1	1	1	1	1					1				1	1	1	1	1	1	1	1		
Zea mexicana (Schrad.) Kuntze								1			1				1																						
Zea sp.										1		1																				1					
Zizania aquatica L.									1			1																									
Zizania latifolia (Griseb.) Hance ex F.Muell.						1						1																				1					
Zizania palustris L.									1									1																			
Zizania sp.								1																					1								
Zizaniopsis miliacea (Michx.) Doell and Aschers.									1																												
Pontederiaceae																																					
Monochoria vaginalis (Burm.F.) C.Presl ex Kunth																								1													

Typhaceae																																					
Typha angustifolia L.																																1					
Typha domingensis Pers.																												1				1	1				
Typha latifolia L.												1																									
Typha sp.																		1															1				
Zingiberaceae																																					
Curcuma longa L.																1																					
Total	3	10	12	9	2	17	8	34	6	32	10	63	13	6	6	83	35	36	35	10	3[2]	1	1	60	14	6	1	14	58	10	8	86	29	11	9	7	25
Crops	1	4	6	5	2	5	6	8	3	7	4	16	4	3	3	8	20	7	8	4	1	1	1	6	4	2	1	5	7	7	2	15	6	4	4	3	3
Grasses (Poaceae)	3	10	12	8	2	16	8	34	6	30	10	53	13	6	6	75	21	17	31	10	3	1	1	47	11	2	1	13	55	10	8	73	20	11	9	5	24
Other monocots (Araceae, Cannaceae, Cyperaceae, Pontederiaceae, Typhaceae, Zingiberaceae)	0	0	0	1	0	1	0	0	0	2	0	4	0	0	0	8	2	18	3	0	0	0	0	12	3	4	0	1	3	0	0	11	9	0	0	2	1
Eudicots (Amaranthaceae, Asteraceae, Brassicaceae, Euphorbiaceae, Fabaceae, Pedaliaceae, Polygonaceae, Solanaceae)	0	0	0	0	0	0	0	0	0	0	0	6	0	0	0	0	12	1	0	0	0	0	0	1	0	0	0	0	0	0	0	2	0	0	0	0	0

1: Green indicates associated hosts; yellow indicates associated crop hosts; and brown indicates potential trap plants

2: *R. albinella* has been reported from two wild grasses, but the original report has not been located for this table

Table S3 Data sources for Table S2

Stemborer Species	Source Information on Host Associations
Ancylolomia chrysographella	[2]
Bathytricha truncata	[73]
Busseola fusca	[2]
Chilo agamemnon	[3,74]
Chilo aleniellus	[75-77]
Chilo auricilius	[2,78-81]
Chilo diffusilineus	[11,17,19]
Chilo partellus	[2,11,74,82-87]
Chilo plejadellus	[2]
Chilo polychrysus	[2,88,89]
Chilo sacchariphagus indicus	[2]
Chilo suppressalis	[2,74,89-98]*
Chilo zacconius	[2,9,76,99-101]
Coniesta ignefusalis	[2]
Diatraea lineolata	[2]
Diatraea saccharalis	[2,61,64,102,103]
Elasmopalpus lignosellus	[2,104]
Eldana saccharina	[2,3,11,17,18,76,105]
Eoreuma loftini	[2,106-115]
Maliarpha separatella	[2,9,100]
Rupela albinella	[64,116]
Scirpophaga fusciflua	[52,117]
Scirpophaga gilviberbis	[19]
Scirpophaga incertulas	[2,19,89,95,118-126]*
Scirpophaga innotata	[2,126-128]
Scirpophaga nivella	[89,129]
Scirpophaga virginia	[52,57]
Sesamia botanephaga	[2,3,11,17,130]
Sesamia calamistis	[2,11,17,99,131]
Sesamia cretica	[2]
Sesamia epunctifera	[2,132]
Sesamia inferens	[2,89,133]
Sesamia nonagrioides (botanephaga)	[2,17,26]
Sesamia penniseti	[2]
Sesamia uniformis	[2]
Diopsis apicalis	[36,134]
Diopsis macrophthalma	[2,36,134]

Table S4 Bionomic data for key rice stemborers

Species	Cluster Size (Eggs)	Egg Duration (Days)	Larval Duration (Days)	Pupal Duration (Days)	Instars (Number)	Prepupal Length (mm)	Pupal Length (mm)	Pupal Weight (mg)	Adult Wingspan (mm)	Lifetime Fecundity (Eggs)	Sources
Africa											
C. agamemnon			20-27					40-45	16-19		[25,41]
C. diffusilineus									8-13		[101]
C. partellus	10-100	7-8 at 22°C; 3-4 at 30°C	40-42 at 22°C; 17-21 at 30°C	4-10; 11-12.5 at 22°C; 5.5-7 at 30°C	5-6			95-145 ♀; 45-75 ♂	20-25	250-340 realized, 550-700 potential at 22°C; 340-425 realized, 750-865 potential at 30°C	[11,101,135-137]
C. zacconius	12-135	4-6	22-28	5-8	5-6	11-12	11-12		11-14		[100,101]
E. saccharina		5-6 at 28°C	30-33 at 28°C	10 at 28°C	6			160-185 ♀, 108-112 ♂		270-360; 760-990	[138]
M. separatella		4.2d at 32.1°C	30-50; 18.8 at 31.9°C		5-7		20			65-310	[31,100,139,140]
S. calamisis	100; < 20	6-9	27-50; 35-36 at 28°C	7-13		20-40	17			300-350	[11,17,31,100]
S. nonagrioides (botanephaga)	up to 100	9 at 21°C, 5 at 30°C	50 at 21°C, 30 at 30°C	13 at 21°C, 10 at 30°C	5-6	30-40		200-250 ♀; 110-160 ♂	30-40	100-550	[26,141-144]
Americas											
C. plejadellus	10-30	5							25-40 ♀; 9-15 ♂		[101]
D. saccharalis	5-50	4-9	25-35	7-11	5-6, up to 14 during extended development		16-20	75-116 ♀; 55-67 ♂;	27-39 ♀; 18-28 ♂	150-750	[64]
E. lignosellus		4.4 at 21°C; 2.2 at 30°C	29.5 at 21°C;' 18.1 at 30°C	11.8 at 21°C; 6.6 at 30°C	5-9	16.2	8	26-28	17-22	200-420; 56 at 21°C; 165 at 30°C	[104,145,146]
E. loftini	<100	14 at 20°C; 5 at 32°C	78 at 20°C; 21 at 32°C	21 at 20°C; 7d at 32°C	5 ♂, 6 ♀	19-25	16-20	26 ♀; 18 ♂		350-400	[112,113]

R. albinella	30-250	6-10		28-36	5	30-35	20-25	55-65 ♂; 100-145 ♀	35-45 ♀; 23-24 ♂	250-800	[64,147]
Asia											
B. truncata				12 at 25°C		25-40	19		40		[148]
C. auricilius		5.8-8.8	21-85	5.8-14	5-8	25-30	13-14 ♀		8-13	90-220	[78,89,101,149,150]
C. polychrysus	30-200	6		4		21-25	12-20.5 ♀, 10-14.5 ♂		16-25 ♀; 6.7-7.5 ♂	488	[68,89,101,150]
C. suppressalis	40-110 - depends on generation	11.5 at 20°C; 5.5 at 32°C	80 at 20°C; 55 at 22°C; 24 at 31°C; 27 at 32°C	13 at 20°C; 9 ♂, 12 ♀ at 22°C; 6 ♂, 6 ♀ at 31°C; 5.5 at 32°C	5-8		15-18.5 ♀; 13-15.5 ♂	44-52 ♀, up to 70 under warm conditions; 39-45 ♂	20-30 ♀, 11-15 ♂	100-550	[55,89,150-155]
S. fusciflua	64-212	5-7		7-9	5				21-27 ♀; 16-22 ♂	64-212	[156]
S. incertulas	40-110 - depends on generation	17 at 20°C; 6 at 32°C	81d at 20°C; 21 at 32°C	26 at 20°C; 8 at 32°C	5-8	17-17.5	14.5-18 ♀; 11.5-15 ♂	30-65 ♀; 8-10 ♂	24-36	220	[55,89,150,151,155,157-161]
S. inferens	30-160	7-8 at 24°C; 4-9 in summer, 9-25 in winter	21-28	5-36 5-12 in summer	5-7	26-35	17-20 ♀; 14-16 ♂		33	300-600	[89,150,162]
S. innotata	70-260	4-9	19-31	7-11		25	14.5-19.5 ♀; 12.5-14.5 ♂				[68,150,163]
S. nivella		8	26	12					14-40		[164]
S. virginia									15-16		[57]

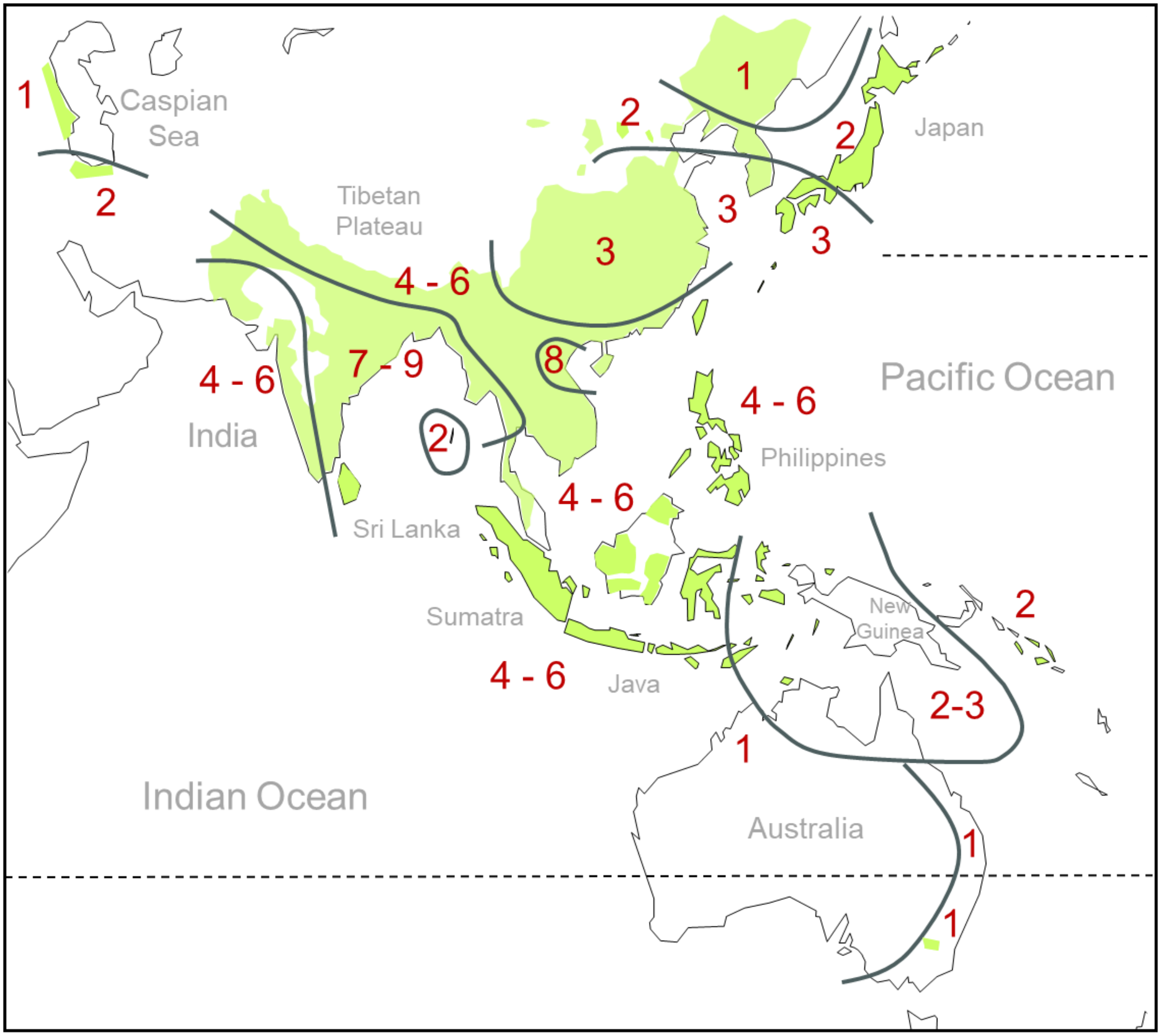

Figure S1 Species richness of stemborer assemblages attacking rice in Asia. The green area indicates the main rice growing regions of Asia, including around the Caspian Sea. Species richness is based on distributions presented in Figure 1 and in the source information cited in Table S1.

References

1. Maes, K.V.N. Revision of the genus *Adelpherupa* Hampson (Lepidoptera: Pyraloidea, Crambidae, Schoenobiinae) with the description of five new species. *Journal of Natural History* **2002**, *36*, 1707-1724. https://doi.org/10.1080/00222930110061878.
2. Khan, Z.R.; Litsinger, J.A.; Barrion, A.T.; Villanueva, F.F.D.; Fernandez, N.J.; Taylo, L.D. *World Bibliography of Rice Stem Borers 1794–1990*; International Rice Research Institute: Los Baños, Philippines: 1991.
3. Metwally, M. Ecological studies on lepidopterous stem borers associated with the main graminaceous weeds and crops at Kafr El-Sheikh region. *Journal of Plant Protection and Pathology* **2010**, *1*, 319-330.
4. Melamed-Madjar, V. Status of *Chilo agamemnon* Bles. in Israel and the probable reasons for the decrease in its populations. *International Journal of Tropical Insect Science* **1990**, *11*, 541-545. https://doi.org/10.1017/S1742758400021093.
5. Gounou, S.; Schulthess, F. Effect of traditional rice / maize intercropping on population densities, crop damage and parasitism of stem-borers in the Ivory Coast. *African Plant Protection* **2006**, *12*, 93-102. https://doi.org/10.10520/EJC87793.
6. Schulten, G.; Feijen, H. A new species of *Trichogramma* (Hymenoptera: Trichogrammatidae) from Malawi, parasitizing eggs of *Chilo diffusilineus* (de Joannis). *Entomologische Berichten. Deel* **1982**, *42*, 142-144.
7. Vercambre, B.; Bordat, D.; Djiba, S. Occurrence and distinction of the two principal species of *Chilo* in rice, in Casamance, Senegal. *Agron. Trop.* **1990**, *45*, 131–138.
8. Djiba, S. Le devenir de *Chilo* spp. (Lepido., Pyralidae) en saison sèche dans la région de Casamance. *Sahel IPM* **1995**, *2*, 18–21.
9. Ba, N.M.; Dakouo, D.; Nacro, S.; Karamage, F. Seasonal abundance of lepidopteran stemborers and diopsid flies in irrigated fields of cultivated (*Oryza sativa*) and wild rice (*Oryza longistaminata*) in western Burkina Faso. *International Journal of Tropical Insect Science* **2008**, *28*, 30-36.
10. Akinsola, E.; Agyen-Sampong, M. The ecology, bionomics and control of rice stem-borers in West Africa. *International Journal of Tropical Insect Science* **1984**, *5*, 69-77.
11. Ingram, W. The lepidopterous stalk borers associated with Gramineae in Uganda. *Bulletin of Entomological Research* **1958**, *49*, 367-383.
12. Ho, D.T.; Njokah, J.J.; Kibuka, J.G. Studies on rice stemborers in Kenya with emphasis on *Maliarpha separatella* Rag. *Insect Science and Its Application* **1983**, *4*, 65-73. https://doi.org/10.1017/s1742758400004045.
13. Leonard, A.; Rwegasira, G.M. Abundance and spatial dispersion of rice stem borer species in Kahama, Tanzania. *Journal of Insect Science* **2015**, *15*, 1-5.
14. Adewoye, B.B.; Anikwe, J.C.; Makanjuola, W.A. Incidence and relative abundance of rice stem borers in three selected rice fields in Lagos and Ogun States, Nigeria. *Bio-Research* **2021**, *19*, 1258-1269.
15. Togola, A.; Nwilene, F.E.; Agbaka, A.; Anato, F.; Agunbiade, T.A.; Chougourou, D.C. Farmer knowledge of rice stem borers and their damage in various ecological zones of Benin (West Africa). *Cahiers Agricultures* **2010**, *19*, 262-266, doi:10.1684/agr.2010.0410.
16. Alam, M.S. A survey of rice pests in Nigeria. *Tropical Pest Management* **1992**, *38*, 115-118.
17. Harris, K.M. Lepidopterous stem borers of cereals in Nigeria. *Bulletin of Entomological Research* **1962**, *53*, 139-171, doi:10.1017/S0007485300048021.
18. Atkinson, P.R. Distribution and natural hosts of *Eldana saccharina* Walker in Natal, its oviposition sites and feeding pattern. *Proceedings of the South African Sugar Technologist's Association* **1979**, *53*, 111-115.
19. Lewvanich, A. A revision of the old world species of the *Scirpophaga complex* (Lepidoptera: Pyralidae): a taxonomic and zoogeographic study, with a discussion on their affinities. University of London, London, UK, 1971.
20. Ahmad, I.; Afzal, M.; Khan, A.M.M. Aspects of larval morphology and larval and adult key to the rice stem borers (Insecta: Lepidoptera) and a new record of a rice stem borer *Niphadoses gilviberbis* (Zell.) from Pakistan *Pak. J. Sci. Ind. Res.* **1979**, *23*, 40-45.
21. Na, B.H.M.P. Etude morphologique et biologique de *Scirpophaga melanoclista-* (Lep., Pyralidae, Schoenobiinae), un ravageur du riz -irrigué en Côte d'Ivoire. Morphologie et biolo.gie d'un parasite d'œuf: *Telenomus thestor* NIXON (Hym., Scelionidae). Ph.D. Thesis, l'Universite Pierre et Marie Curie, Paris, France,1978.
22. Monnet, C. The control of rice insects in the Ivory Coast. In Proceedings of the Congress on the Control of Insects in the Tropical Environment, Part I, Tropical Crops, 13–16 March 1979; Chamber of Commerce and Industry of Marseilles; Marseilles, France, 1979; pp. 559-567.
23. Emosairue, S.; Shiyam, J. A compendium of insect pests and natural enemies associated with lowland rice in south eastern Nigeria. *Global Journal of Pure and Applied Sciences* **2000**, *6*, 385-388.
24. Calatayud, P.-A.; Le Ru, B.P.; Van den Berg, J.; Schulthess, F. Ecology of the African Maize Stalk Borer, Busseola fusca (Lepidoptera: Noctuidae) with Special Reference to Insect-Plant Interactions. *Insects* **2014**, *5*, 539-563, doi:10.3390/insects5030539.
25. Hafez, M.; Salama, H.S.; Tolba, R.A. Investigations on the biology of the corn borer *Sesamia cretica* Led.(Lepidoptera—Agrotidae). *Zeitschrift für Angewandte Entomologie* **1971**, *67*, 38-44.

26. Camargo, A.M.; Arias-Martin, M.; Castanera, P.; Farinos, G.P. Performance of *Sesamia nonagrioides* on cultivated and wild host plants: Implications for Bt maize resistance management. *Pest Management Science* **2020**, *76*, 3657-3666. https://doi.org/10.1002/ps.5913.
27. Ntanos, D.A.; Koutroubas, S.D. Evaluation of rice for resistance to pink stem borer (Sesamia nonagrioides Lefebre). *Field Crops Research* **2000**, *66*, 63-71. https://doi.org/10.1016/S0378-4290(00)00063-0.
28. El-Hawary, I.S.; El-Naggar, M.A.; Henishen, E.Z. Rice stem borer, *Chilo agamemnon*, population fluctuation and rice varietal resistance. *Journal of Plant Protection and Pathology of Mansoura University* **2015**, *6*, 1519-1525.
29. John, V.T.; Alum, M.S.; Thottappilly, G. Diseases and insect pests of wetland rice in tropical Africa. In The Wetlands and Rice in SubSaharan Africa. Proceedings of an International Conference on Wetland Utilization for Rice Production in SubSaharan Africa, 4~8 November 1985 Juo, A.S.R., Lowe, J.A., Eds.; International Institute of Tropical Agriculture, Ibadan, 1986; pp. 141-150.
30. Nye, I.W.B. The insect pests of graminaceous crops in East Africa. Report of a survey carried out between March 1956 and April 1958. *The insect pests of graminaceous crops in East Africa. Report of a survey carried out between March 1956 and April 1958. Colonial Research Studies Number 31*; Colonial Office, London, UK, **1960**.
31. Heinrichs, E.A.; Barrion, A.T. *Rice-feeding insects and selected natural enemies in West Africa: biology, ecology, identification*; International Rice Research Institute: Los Baños, Philippines, 2004.
32. Umeh, E.D.N.; Joshi, R.C.; Ukwungwu, M.N. Biology, status and management of rice insect pests in Nigeria. *Crop Protection* **1992**, *11*, 408-413, doi:10.1016/0261-2194(92)90022-w.
33. Pollet, A. Pests on rice in Ivory Coast. 5. Interrelationship between *Maliarpha separatella* and *Pyricularia oryzae Journal of Applied Entomology* **1978**, *85*, 324-327.
34. Bianchi, G.; Rasoloarison, B.; Genini, M. Noxiousness of the African white stem borer *Maliarha separatella* Rag (Pyralidae, Phycitinae) in irrigated paddy fields at Lake Alaotra (Madagascar). *Insect Science and its Application* **1993**, *14*, 667-673. https://doi.org/10.1017/s1742758400018099.
35. Bocco, R.; Elie, D.; Gandonou, C. Diopsids (*Diopsis thoracica* and *D. apicalis*) damaging rice production in Africa: A Review. *International Journal of Current Research. Bioscience and Plant Biology* **2017**, *4*, 33–41.
36. Chiasson, H.; Hill, S. Population density, development and behaviour of *Diopsis longicornis* and *D. apicalis* (Diptera: Diopsidae) on rice in the Republic of Guinee. *Bulletin of Entomological Research* **1993**, *83*, 5-13.
37. Descamps, M. Contributions to the study of Diptera: Diopsidae pest of rice in North Cameroon. *J. Agric. Trop. Bot. Appl.* **1957**, *4*, 83-93.
38. Bernard, V. *Diopsis thoracica* WEST (Dipt. Diopsidae) important ravageur du riz en Afrique de l'Ouest : données bio-écologiques et application à la lutte intégrée. . *L'Agronomie Tropicale* **1975**, *37*, 89–98.
39. Feijen, H.R.; Feijen, C. A new species of *Diopsis* L. (Diptera: Diopsidae) from South Africa and Swaziland, and brief review of African species with a large apical wing spot. *African Invertebrates* **2012**, *53*, 125-142. https://doi.org/10.10520/EJC121923.
40. Gautam, R.D.; Suroshe, S.; Mahapatro, G.K. Unique record of chloropid pest in rice (*Oryza sativa*). *Indian Journal of Agricultural Sciences* **2009**, *79*, 841-843.
41. Taneja, S.; Nwanze, K.F. Mass rearing of *Chilo* spp. on artificial diets and its use in resistance screening. *International Journal of Tropical Insect Science* **1990**, *11*, 605-616.
42. Chu, Y.I. Ecology of rice borers in Taiwan. In Proceedings of the Symposium on Rice Insects, July 1971, Tokyo, Japan, Tropical Agriculture Research Centre, |Ministry of Agriculture and Environment: Tokyo, Japan; 1971; pp. 155-162.
43. Chakravorty, S. Damage to rice grains by stem borer attack. *International Rice Research Newsletter* **1979**, *4*, 1.
44. Catling, H.; Islam, Z.; Pattrasudhi, R. Seasonal occurrence of the yellow stem borer *Scirpophaga incertulas* (Walker) on deepwater rice in Bangladesh and Thailand. *Agriculture, Ecosystems and Environment* **1984**, *12*, 47-71.
45. Rahman, M.T.; Khalequzzaman, M.; Khan, M.A.R. Assessment of infestation and yield loss by stem borers on variety of rice. *Journal of Asia-Pacific Entomology* **2004**, *7*, 89-95.
46. Khari, N.A.M.; Ab Hamid, S. Abundance and infestation of Rice Stem Borer in North Malaysia. *Malaysian Applied Biology* **2022**, *51*, 165-177.
47. Razali, R.; Yaakop, S.; Abdullah, M.; Ghazali, S.Z.; Zuki, A.A. Insect species composition in an under SRI management in Tanjung Karang, Selangor, Malaysia. *Malaysian Applied Biology* **2015**, *44*, 59-66.
48. Ooi, A.C. A padi stem-borer survey in the Muda scheme, Kedah. *Malaysian Agricultural Journal* **1974**, *49*, 525-531.
49. Jiang, M.X.; Cheng, J.A. Interactions between the striped stem borer *Chilo suppressalis* (Walk.) (Lep., Pyralidae) larvae and rice plants in response to nitrogen fertilization. *Journal of Pest Science* **2003**, *76*, 124-128. https://doi.org/10.1007/s10340-003-0001-x.
50. Koch, L. Rice stem borers at the Kimberley Research Station. *Journal of the Department of Agriculture, Western Australia, Series 4* **1960**, *1*, 1061-1063.
51. Kumar, A.; Ram, L.; Singh, R.; Singh, B. Biology and behavior of white stem borer (*Scirpophaga fusciflua*) on rice (*Oryza sativa*) in India. *Indian J. Agric. Sci.* **2018**, *88*, 1937–1940.
52. Saini, V.; Singh, S.; Rawal, R.; Venkatesh, Y. Species diversity and distribution of *Cnaphalocrocis* and *Scirpophaga* (Lepidoptera: Crambidae) species complex in rice in Tamil Nadu, India. *J. Entomol. Zool. Stud.* **2017**, *5*, 1308–1313.

53. Tandon, V.; Srivastava, A. Seasonal incidence of white stemborer, *Scirpophaga fasciflua* Hampson on paddy in Himachal Pradesh. *Oryza* **2018**, *55*, 349-352.
54. Tandon, V.; Srivastava, A. Assessment of Yield Losses Due to White Stem Borer *Scirpophaga fusciflua* (Hampson) in Rice. *Indian Journal of Entomology* **2022**, 634-636.
55. Horgan, F.G.; Romena, A.M.; Bernal, C.C.; Almazan, M.L.P.; Ramal, A.F. Stem borers revisited: Host resistance, tolerance, and vulnerability determine levels of field damage from a complex of Asian rice stemborers. *Crop Protection* **2021**, *142*, 105513, doi:10.1016/j.cropro.2020.105513.
56. Litsinger, J.A.; Alviola, A.L.; Dela Cruz, C.G.; Canapi, B.L.; Batay-An, E.H.; Barrion, A.T. Rice white stemborer *Scirpophaga innotata* (Walker) in southern Mindanao, Philippines. II. Synchrony of planting and natural enemies. *Int. J. Pest Manag.* **2006**, *52*, 23-37. https//doi.org/10.1080/09670870600552463.
57. Saini, V.; Ramaraju, K.; Chitra, N. Occurrence of new stem borer species, *Scirpophaga virginia* Lepidoptera: Pyraloidea: Crambidae from Tamil Nadu, India and its taxonomic re-description. *Ecology, Environment and Conservation* **2017**, *23*, 325-328.
58. Department of Primary Industries. *Rice field guide to pests, diseases and weeds in southern New South Wales*; Department of Primary Industries, Yanco, NSW: Australia, 2013.
59. Litsinger, J.A.; Barrion, A.T.; Canapi, B.L.; Lumaban, M.D.; dela Cruz, C.G.; Pantua, P.C. Philippines rice stemborers: a review. *Philipp. Ent.* **2011**, *25*, 1-47.
60. Oliver, B.; Gifford, J. Weight differences among stalk borer larvae collected from rice lines showing resistance in field studies. *Journal of Economic Entomology* **1975**, *68*, 134-134.
61. Joyce, A.L.; Chicas, M.S.; Cervantes, L.S.; Paniagua, M.; Scheffer, S.J.; Solis, M.A. Host-plant associated genetic divergence of two *Diatraea* spp. (Lepidoptera: Crambidae) stemborers on novel crop plants. *Ecology and Evolution* **2016**, *6*, 8632-8644. https//doi.org/10.1002/ece3.2541.
62. Roldan, E.L.; Beuzelin, J.M.; Vanweelden, M.T.; Cherry, R.H. The Sugarcane Borer (Lepidoptera: Crambidae) infests rice at low population levels in Florida. *Journal of Economic Entomology* **2020**, *113*, 538-542. https//doi.org/10.1093/jee/toz255.
63. Wilson, B.E.; Villegas, J.M.; Stout, M.J.; Landry, K.J. Relative yield loss from stem borers (Lepidoptera: Crambidae) and Rice Water Weevil (Coleoptera: Curculionidae) in rice. *Journal of Economic Entomology* **2021**, *114*, 1159-1165. https//doi.org/10.1093/jee/toab046.
64. Hummelen, P.J. *Relations between two rice borers in Surinam, Rupela albinella (Cr.) and Diatraea saccharalis (F.), and their hymenopterous larval parasites*; Veenman and Zonen: Wageningen, Netherlands, 1974.
65. Pantoja, A.; Matta, J.; Correa, F. Stem borer, *Rupela albinella* (Cramer), and stem-rot, *Sclerotium oryza*, in southwestren Colombioa rice fields. *Journal of Agriculture of the University of Puerto Rico* **1994**, *78*, 17-22.
66. Ferreira, E.; Barrigossi, J.A.F. A field technique for infesting rice with *Elasmopalpus lignosellus* (Zeller)(Lepidoptera: Pyralidae) and evaluating insecticide treatments. *Neotropical Entomology* **2003**, *32*, 367-371.
67. Rebelles, R. Principle pests of upland rice. *Informe Agropecuário (Belo Horizonte)* **1989**, *14*, 44-58.
68. Rothschild, G. The biology and ecology of rice-stem borers in Sarawak (Malaysian Borneo). *Journal of Applied Ecology* **1971**, 287-322.
69. Cook, M. Revision of the genus *Maliarpha* (Lepidoptera: Pyralidae), based on adult morphology with description of three new species. *Bulletin of Entomological Research* **1997**, *87*, 25-36. https//doi.org/10.1017/s0007485300036324.
70. Li, C.S. Sugarcane insect pests with special reference to the moth borers in the Markham Valley, Papua New Guinea. *Mushi* **1985**, *50*, 13-18.
71. Sandhu, G.; Chander, R. Occurrence of green striped borer, *Maliarpha separatella* Ragonot on sorghum in the Punjab. *Journal of the Bombay Natural History Society* **1976**, *72*, 872-873.
72. Delfosse, E. Les mouches de la famille des Diopsidae (Insecta: Diptera). *Le Bulletin d'Arthropoda* **2006**, *27*, 31-38.
73. Butterfly House. Lepidoptera. Available online: (http://lepidoptera.butterflyhouse.com.au/acro/truncata.html) (accessed on 15 June 2023).
74. Mohyuddin, A.I. Biological control of *Chilo* spp. in maize, sorghum and millet. *International Journal of Tropical Insect Science* **1990**, *11*, 719-732. https//doi.org/10.1017/S1742758400021287.
75. Akinsola, E. Management of *Chilo* spp. in rice in Africa. *International Journal of Tropical Insect Science* **1990**, *11*, 815-823.
76. Kfir, R.; Overholt, W.A.; Khan, Z.R.; Polaszek, A. Biology and management of economically important lepidopteran cereal stem borers in Africa. *Annual Review of Entomology* **2002**, *47*, 701-731. https//doi.org/10.1146/annurev.ento.47.091201.145254.
77. Moyal, P.; Tran, M. *Chilo aleniellus* (Lepidoptera: Pyralidae), a stem borer of maize in Côte d'Ivoire. *Bulletin of Entomological Research* **1992**, *82*, 67-72. https//doi.org/10.1017/S0007485300051506.
78. Huang, R.; Huang, P.; Xiong, C. Studies on the occurrence of *Chilo auricilis* Dudgeon in Yibing Prefecture, Shichuan. *Insect Knowledge* **1985**, *22*, 104-106.
79. Huang, Q.; Yang, J.; Ling, Y.; Jiang, X.; Yu, X.; Rong, Y.; Mo, Y.; Wang, G.; Fu, C.; Long, L. Feeding and oviposition preferences of *Chilo auricilius* Dudgeon to four species of host plants. *Journal of Southern Agriculture* **2016**, *47*, 55-58.

80. Yi, X.; Shi, S.; Wang, P.; Chen, Y.; Lu, Q.; Wang, T.; Zhou, X.; Zhong, G. Characterizing potential repelling volatiles for "push-pull" strategy against stem borer: a case study in *Chilo auricilius*. *BMC Genomics* **2019**, *20*, 751. https//doi.org/10.1186/s12864-019-6112-4.
81. Catindig, J.; Barrion, A.; Litsinger, J. Life history, alternate hosts, and natural enemies of the gold fringed borer, *Chilo auricilius* Dudgeon (Lepidoptera: Pyralidae): a new pest of upland rice (*Oryza sativa*) in the Philippines. In Proceedings of the Proceedings of the 17th Anniversary and Annual Convention of the Pest Control Council of the Philippines, Iloilo City (Philippines), 8-10 May 1986, 1986.
82. January, B.; Rwegasira, G.M.; Tefera, T. Rice stem borer species in Tanzania: a review. *The Journal of Basic and Applied Zoology* **2020**, *81*, 1-9.
83. van den Berg, J. Vetiver grass (*Vetiveria zizanioides* (L.) Nash) as trap plant for *Chilo partellus* (Swinhoe) (Lepidoptera : Pyralidae) and *Busseola fusca* (Fuller) (Lepidoptera : Noctuidae). *Annales De La Societe Entomologique De France* **2006**, *42*, 449-454. https//doi.org/10.1080/00379271.2006.10697478.
84. Rebe, M.; Van den Berg, J.; McGeoch, M. Growth and development of *Chilo partellus* (Swinhoe)(Lepidoptera: Crambidae) on cultivated and indigenous graminaceous host plants. *African Entomology* **2004**, *12*, 253-258.
85. Mohamed, H.; Khan, Z.; Overholt, W.; Elizabeth, D. Behaviour and biology of *Chilo partellus* (Lepidoptera: Pyralidae) on maize and wild gramineous plants. *International Journal of Tropical Insect Science* **2004**, *24*, 287-297.
86. Chamberlain, K.; Khan, Z.R.; Pickett, J.A.; Toshova, T.; Wadhams, L.J. Diel periodicity in the production of green leaf volatiles by wild and cultivated host plants of stemborer moths, *Chilo partellus* and *Busseola fusca*. *Journal of Chemical Ecology* **2006**, *32*, 565-577. https//doi.org/10.1007/s10886-005-9016-5.
87. Midega, C.A.O.; Khan, Z.R.; Pickett, J.A.; Nylin, S. Host plant selection behaviour of *Chilo partellus* and its implication for effectiveness of a trap crop. *Entomologia Experimentalis et Applicata* **2011**, *138*, 40-47. https//doi.org/https://doi.org/10.1111/j.1570-7458.2010.01073.x.
88. Chaudhary, R.; Khush, G.; Heinrichs, E. Varietal resistance to rice stem-borers in Asia. *International Journal of Tropical Insect Science* **1984**, *5*, 447-463.
89. Katti, G.; Shanker, C.; Padmakumari, A.P.; Pasalu, I.C. *Rice stem borers in India: Species composition and distribution*; Directorate of Rice Research: Hyderabad, India, 2011.
90. Huang, C.; Hu, B.; Li, J.H.; Wang, Y.M. Water-oats harbors two strains of the striped stem borer *Chilo suppressalis* (Lepidoptera: Crambidae) with temporal divergence in mating behavior. *Applied Entomology and Zoology* **2016**, *51*, 457-463, doi:10.1007/s13355-016-0421-8.
91. Quan, W.L.; Liu, W.; Zhou, R.Q.; Chen, R.; Ma, W.H.; Lei, C.L.; Wang, X.P. Difference in diel mating time contributes to assortative mating between host plant-associated populations of *Chilo suppressalis*. *Scientific Reports* **2017**, *7*. https//doi.org/10.1038/srep45265.
92. Jiang, W.H.; Li, H.D.; Cheng, X.F.; Ye, J.R.; Feng, Y.B.; Han, Z.J. Study on host plants for reproduction of *Chilo suppressalis*. *Journal of Asia-Pacific Entomology* **2015**, *18*, 591-595. https//doi.org/10.1016/j.aspen.2015.07.009.
93. Hou, M.L.; Hao, L.X.; Han, Y.Q.; Liao, X.L. Host status of wheat and corn for *Chilo suppressalis* (Lepidoptera: Crambidae). *Environmental Entomology* **2010**, *39*, 1929-1935. https//doi.org/10.1603/en10081.
94. Kung, K.-S. Ecological studies on the rice stem borer (*Chilo suppressalis* Walker) in Taiwan (II): Host plant survey. In Proceedings of the Proceedings of a Symposium on Tropical Agriculture Researches, 19-24 July, 1971, Tokyo, Japan, 1971; p. 27.
95. Morrill, W.L.; Arida, G.S. Oviposition preference and survival of selected rice insects on wheat. *Journal of Economic Entomology* **1991**, *84*, 656-658. https//doi.org/10.1093/jee/84.2.656.
96. Cuong, N.L.; Cohen, M.B. Field survey and greenhouse evaluation of non-rice host plants of the striped stem borer, *Chilo suppressalis* (Lepidoptera : Pyralidae), as refuges for resistance management of rice transformed with *Bacillus thuringiensis* toxin genes. *Bulletin of Entomological Research* **2002**, *92*, 265-268. https//doi.org/10.1079/ber2002163.
97. Zheng, X.; Xu, H.; Chen, G.; Wu, J.; Lu, Z. Potential function of Sudan grass and vetiver grass as trap crops for suppressing population of stripped stem borer, *Chilo suppressalis* in rice. *Chinese Journal of Biological Control* **2009**, *25*, 299-303.
98. Lu, Y.H.; Zheng, X.S.; Lu, Z.X. Application of vetiver grass *Vetiveria zizanioides*: Poaceae (L.) as a trap plant for rice stem borer *Chilo suppressalis*: Crambidae (Walker) in the paddy fields. *Journal of Integrative Agriculture* **2019**, *18*, 797-804. https//doi.org/10.1016/s2095-3119(18)62088-x.
99. Sampson, M.; Kumar, R. Alternative host plants of sugar-cane stem-borers in southern Ghana. *International Journal of Tropical Insect Science* **1986**, *7*, 539-541.
100. Wopereis, M.C.S.; Defoer, T.; Idinoba, P.; S., D.; Dugué, M.J. *Participatory learning and action research (PLAR) for integrated rice management (IRM) in inland valleys of Sub-Saharan Africa: Technical manual*; Africa Rice Center: AfricaRice Training Series. Cotonou, Benin, 2009.
101. Sallam, M.S.; Allsopp, P.G. *Preparedness for borer incursion*; Bureau of Sugar Experiment Stations, Bundaberg, Qld, Australia: 2002.

102. Beuzelin, J.M.; Meszaros, A.; Reagan, T.E.; Wilson, L.T.; Way, M.O.; Blouin, D.C.; Showler, A.T. Seasonal infestations of two stem borers (Lepidoptera: Crambidae) in noncrop grasses of Gulf Coast rice agroecosystems. *Environmental Entomology* **2011**, *40*, 1036-1050. https//doi.org/10.1603/en11044.
103. White, W.; Richard, R.; Hale, A. *Erianthus*: a sugarcane relative with potential as a source of resistance to the stem borer *Diatraea saccharalis* (F.)(Lepidoptera: Crambidae). In Proceedings of the International Society of Sugar Cane Technologists: Proceedings of the XXVIIIth Congress, June 24 to June 27, 2013, São Paulo, Brazil, 2013; pp. 825-834.
104. Gill, H.K.; Capinera, J.L.; McSorley, R. *Lesser Cornstalk Borer, Elasmopalpus lignosellus (Zeller)(Insecta: Lepidoptera: Pyralidae). Document EENY-155*, Institute of Food and Agricultural Sciences - Extension, University of Florida; Gainesville, FL, USA, 2011. https://entnemdept.ufl.edu/creatures/field/lesser_cornstalk_borer.htm (accessed on 15 June 2023).
105. Atachi, P.; Sekloka, E.; Schulthess, F. Study on some bioecological aspects of *Eldana saccharina* Walker (Lep., Pyralidae) on *Zea mays* L. and alternative host plants. *Journal of Applied Entomology* **2005**, *129*, 447-455.
106. Showler, A.T.; Beuzelin, J.M.; Reagan, T.E. Alternate crop and weed host plant oviposition preferences by the Mexican rice borer (Lepidoptera: Crambidae). *Crop Protection* **2011**, *30*, 895-901. https//doi.org/10.1016/j.cropro.2011.02.006.
107. Showler, A.T.; Reagan, T.E. Mexican rice borer, *Eoreuma loftini* (Dyar)(Lepidoptera: Crambidae): range expansion, biology, ecology, control tactics, and new resistance factors in United States sugarcane. *American Entomologist* **2017**, *63*, 36-51.
108. Showler, A.T.; Wilson, B.E.; Reagan, T.E. Mexican rice borer (Lepidoptera: Crambidae) injury to corn greater than to sorghum and sugarcane under field conditions. *Journal of economic entomology* **2012**, *105*, 1597-1602.
109. Showler, A.T.; Reagan, T.E. New findings on factors affecting Mexican rice borer, *Eoreuma loftini* Dyar, infestation of sugarcane: Leaf characteristics, drought stress, soil fertility, and alternate host plants. *International Sugar Journal* **2011**, *113*, 744-744.
110. Beuzelin, J.M.; Meszaros, A.; Way, M.O.; Reagan, T.E. Rice harvest cutting height and ratoon crop effects on late season and overwintering stem borer (Lepidoptera: Crambidae) infestations. *Crop Protection* **2012**, *34*, 47-55. https//doi.org/10.1016/j.cropro.2011.11.019.
111. Beuzelin, J.M.; Reagan, T.E.; Way, M.O.; Meszaros, A.; Akbar, W.; Wilson, L.T. Potential impact of Mexican rice borer non-crop hosts on sugarcane IPM. *International Sugar Journal* **2011**, *113*, 660-665.
112. Beuzelin, J.M.; Wilson, B.E.; VanWeelden, M.T.; Meszaros, A.; Way, M.O.; Stout, M.J.; Reagan, T.E. Biology and management of the Mexican Rice Borer (Lepidoptera: Crambidae) in rice in the United States. *Journal of Integrated Pest Management* **2016**, *7*, doi:10.1093/jipm/pmw006.
113. Beuzelin, J.M.; Wilson, L.T.; Showler, A.T.; Meszaros, A.; Wilson, B.E.; Way, M.O.; Reagan, T.E. Oviposition and larval development of a stem borer, *Eoreuma loftini*, on rice and non-crop grass hosts. *Entomologia Experimentalis et Applicata* **2013**, *146*, 332-346. https//doi.org/10.1111/eea.12031.
114. Reay-Jones, F.P.F.; Wilson, L.T.; Showler, A.T.; Reagan, T.E.; Way, M.O. Role of oviposition preference in an invasive crambid impacting two Graminaceous host crops. *Environmental Entomology* **2007**, *36*, 938-951. https//doi.org/10.1603/0046-225x(2007)36[938:roopia]2.0.co;2.
115. Rodríguez-del-Bosque, L.A.; Palomo Salas, J.; Mendez Rodriguez, A. Susceptibility of bermudagrass cultivars to *Eoreuma loftini* (Lepidoptera: Pyralidae) in subtropical Mexico. *Florida Entomologist* **1996**, *79*, 188-193. https//doi.org/10.2307/3495816.
116. Passoa, S.; Habeck, D.H. A description of the larva and pupa of *Rupela albinella*, a pest of rice in Latin America (Lepidoptera: Pyralidae: Schoenobiinae). *Fla. Entomol.* **1987**, *70*, 368–375. https://doi.org/10.2307/3495070.
117. Srivastava, A.; Sarao, P.S.; Ram, L.; Salaia, R.; Singh, A. New record of *Scirpophaga fusciflua* Hampson from North India. *New Facets of 21 st Century Plant Breeding* **2012**, *5*, 62.
118. Chen, Y.H.; Romena, A. Feeding patterns of *Scirpophaga incertulas* (Lepidoptera : Crambidae) on wild and cultivated rice during the booting stage. *Environmental Entomology* **2006**, *35*, 1094-1102. https//doi.org/10.1603/0046-225x-35.4.1094.
119. Sutrisno, H. New Host Record of The Yellow Steam Borer Moths, *Scirpophaga incertulas* Shall (Lepidoptera: Pyralidae). *Indonesian Journal of Biology* **2007**, *4*, 80500.
120. Padhi, G.; Prakasa Rao, P.S. Preliminary studies on reaction of some wild rices to infestation by the yellow rice borer *Tryporyza incertulas* Wlk. *Oryza* **1978**, *15*, 99-101.
121. Zhang, Z.Y.; Soulemane, B.; Guo, X.P.; Zhu, C.L.; Xu, H.L. Grain yield and weed-mediated pest incidence in paddy rice crops under controllable irrigation regimes. *Journal of Food Agriculture & Environment* **2012**, *10*, 626-631.
122. Zaheruddeen, S.M.; Rao, P.S. Record of some wild species of rice as potential hosts of the yellow rice borer. *Science and Culture* **1983**, *49*, 207-208.
123. Dutta, S.; Roy, N. Population dynamics and host preference of a major pest, *Scirpophaga incertulas* Walker (Pyralidae: Lepidoptera). *International Journal of Advanced Science and Research* **2018**, *3*, 120-127.
124. Catindig, J.; Barrion, A.; Litsinger, J. Host range of yellow rice borer, brown and whitebacked planthoppers. In Proceedings of the Proceedings of the 19th Pest Control Council of the Philippines, Cebu City (Philippines), 3-7 May 1988, 1988.
125. Zaheruddeen, S.M.; Rao, P.S. Host status of weed flora of rice ecosystem to the yellow rice borer *Scirpophaga incertulas* Walker. *Oryza* **1983**, *20*, 177-179.

126. Arvind, A. Host plants for yellow rice borer (YSB) *Scirpophaga incertulas* and white stem borer (WSB) *Scirpophaga innotata*. *International Rice Research Newsletter* **1987**, *12*, 3.
127. Ranganath, H.R.; Prasad, G.S.; Sheeba. The sedge *Eleocharis dulcis* (Burm. f.) Henschel (Cyperaceae) traps rice white stem borer *Scirpophaga innotata* (Walker) (Lepidoptera : Pyralidae) in Andamans. *Current Science* **2002**, *83*, 371-372.
128. Rajesh, K.M.; Sinu, P.A.; Nasser, M. *Eleocharis dulcis* (Burm.f) as a promising trap plant for the biocontrol of rice white stem borer, *Scirpophaga innotata* (Walker). *Biological Control* **2021**, *160*, 104676. https://doi.org/10.1016/j.biocontrol.2021.104676.
129. Khaliq, A.; Ashfaq, M.; Akram, W.; CHOI, J.K.; LEE, J.J. Effect of plant factors, sugar contents, and control methods on the Top Borer (*Scirpophaga nivella* F.) Infestation in selected varieties of sugarcane. *Entomological Research* **2005**, *35*, 153-160.
130. Goftishu, M. Ecology and genetic diversity of cereal stem borers in cultivated and natural habitats of Ethiopia. Ph.D. Thesis, University of Fort Hare, Alice, South Africa, 2018.
131. Ndemah, R.; Gounou, S.; Schulthess, F. The role of wild grasses in the management of lepidopterous stem-borers on maize in the humid tropics of western Africa. *Bulletin of Entomological Research* **2007**, *92*, 507-519. https//doi.org/10.1079/BER2002196.
132. Moyal, P.; Le RÜ, B.; Van Den Berg, J.; Ratnadass, A.; Cugala, D.; Matama-Kauma, T.; Pallangyo, B.; Conlong, D.; Defabachew, B. Morphological reinforcement, ancient introgressive hybridization and species delimitation in African stem-borer species of the genus *Sesamia* Guenée (Lepidoptera: Noctuidae). *Systematic Entomology* **2011**, *36*, 421-434. https://doi.org/10.1111/j.1365-3113.2011.00570.x.
133. Liu, Z.; Gao, Y.; Luo, J.; Lai, F.; Li, Y.; Fu, Q.; Peng, Y. Evaluating the non-rice host plant species of *Sesamia inferens* (Lepidoptera: Noctuidae) as natural refuges: resistance management of Bt rice. *Environmental entomology* **2011**, *40*, 749-754.
134. Chiasson, H. Determination of key factors affecting the population dynamics of *Diopsis longicornis* and *D. apicalis* (Diptera: Diopsidae), pests of rice in the republic of Guinée, West Africa. Ph.D. Thesis, McGill University, Montréal, QC, Canada,**1990**.
135. Ofomata, V.; Overholt, W.; Lux, S.; Van Huis, A.; Egwuatu, R. Comparative studies on the fecundity, egg survival, larval feeding, and development of *Chilo partellus* and *Chilo orichalcociliellus* (Lepidoptera: Crambidae) on five grasses. *Annals of the Entomological Society of America* **2000**, *93*, 492-499.
136. Kishore Kumar, V.; Sharma, H.C.; Dharma Reddy, K. Antibiosis mechanism of resistance to spotted stem borer, *Chilo partellus* in sorghum, *Sorghum biocolor*. *Crop Protection* **2006**, *25*, 66-72. https://doi.org/10.1016/j.cropro.2005.04.001.
137. Tamiru, A.; Getu, E.; Jembere, B.; Bruce, T. Effect of temperature and relative humidity on the development and fecundity of *Chilo partellus* (Swinhoe)(Lepidoptera: Crambidae). *Bulletin of Entomological Research* **2012**, *102*, 9-15.
138. Ngomane, N.C. Optimization of formulated artificial diets with the addition of sterols and cryoprotectants for effective rearing and fitness of Eldana saccharina Walker (Lepidoptera: Pyralidae). Stellenbosch: Stellenbosch University, 2021.
139. Bianchi, G. Comportement et nuisibilité de *Maliarpha separatella* Ragonot (Lep. Phycitinae) dans les rizières malgaches du lac Alaotra et modélisation de la dynamique de ses populations. ETH Zurich, 1989.
140. Bianchi, G.; Baumgartner, J.; Delucchi, V.; Rahalivavololona, N.; Skillman, S.; Zahner, P. Sampling egg batches of *Maliarpha separatella* Rag (Lep, Pyralidae) in Madagascan rice fields. *Tropical Pest Management* **1989**, *35*, 420-424, doi:10.1080/09670878909371420.
141. Fantinou, A.A.; Perdikis, D.C.; Chatzoglou, C.S. Development of immature stages of *Sesamia nonagrioides* (Lepidoptera : Noctuidae) under alternating and constant temperatures. *Environmental Entomology* **2003**, *32*, 1337-1342. https//doi.org/10.1603/0046-225X-32.6.1337.
142. Fantinou, A.A.; Perdikis, D.C.H.; Zota, K.F. Reproductive responses to photoperiod and temperature by diapausing and nondiapausing populations of *Sesamia nonagrioides* Lef. (Lepidoptera−Noctuidae). *Physiological Entomology* **2004**, *29*, 169-175. https://doi.org/10.1111/j.1365-3032.2004.00381.x.
143. Fantinou, A.A.; Perdikis, D.C.; Stamogiannis, N. Effect of larval crowding on the life history traits of *Sesamia nonagrioides* (Lepidoptera: Noctuidae). *European Journal of Entomology* **2008**, *105*, 625-630.
144. López, C.; Sans, A.; Asin, L.; EizaGuirre, M. Phenological model for *Sesamia nonagrioides* (Lepidoptera: Noctuidae). *Environmental Entomology* **2001**, *30*, 23-30, doi:10.1603/0046-225X-30.1.23.
145. Sandhu, H.S.; Nuessly, G.S.; Webb, S.E.; Cherry, R.H.; Gilbert, R.A. Life Table Studies of Elasmopalpus lignosellus (Lepidoptera: Pyralidae) on Sugarcane. *Environmental Entomology* **2010**, *39*, 2025-2032. https//doi.org/10.1603/en10038.
146. Sandhu, H.S.; Nuessly, G.S.; Webb, S.E.; Cherry, R.H.; Gilbert, R.A. Temperature-dependent reproductive and life table parameters of *Elasmopalpus lignosellus* (Lepidoptera: Pyralidae) on sugarcane. *Florida Entomologist* **2013**, 380-390.
147. Castro Borbor, Á.R. *Altura del daño ocasionado por larvas de Rupela albinella (Cramer), en tres variedades de arroz, bajo tres sistemas de manejo de agua.* Agronomic Engineering Thesis, Universidad Tecnica de Babahoyo, Babahoyo, Ecuador, 2011.
148. Jarvis, E. *Notes on insects damaging sugar cane in Queensland*; Bureau of Sugar Experiment Stations: Brisbane, Australia, 1916; Volume 3, p. 47.
149. Rao, Y.; Rao, Y. Bionomics of the crambid borer, *Chilo auricilius* Dudgeon infesting rice. *Journal of Entomological Research* **1980**, *4*, 68-72.
150. Hattori, I.; Siwi, S. Rice stemborers in Indonesia. *JARQ* **1986**, *20*, 25-30.

151. Kiritani, K. The biology and life cycle of *Chilo suppressalis* (Walker) and *Tryporyza* (*Shoenobius*) *incertulas* (Walker) in temperate-climate areas. In *Proceedings of the Symposium: The Major Insect Pests of the Rice Plant,* International Rice Research Institute *(IRRI), Los Baños, Philippines, July 1964*: IRRI: Los Baños, Philippines, 1967.
152. Ramoneda, J.; Roig, J. Characteristics of the larval development of the rice stem borer, *Chilo suppressalis* WALKER (Lepidoptera:Crambidae) in the Ebro Delta (Northeastern Spain). *Applied Entomology and Zoology* **1993**, *28*, 267-273, doi:10.1303/aez.28.267.
153. Huang, X.L.; Xiao, L.; He, H.M.; Xue, F.S. Effect of rearing conditions on the correlation between larval development time and pupal weight of the rice stem borer, *Chilo suppressalis*. *Ecology and Evolution* **2018**, *8*, 12694-12701. https//doi.org/10.1002/ece3.4697.
154. Xiao, H.J.; Chen, J.H.; Chen, L.Y.; Chen, C.; Wu, S.H. Exposure to mild temperatures decreases overwintering larval survival and post-diapause reproductive potential in the rice stem borer *Chilo suppressalis*. *Journal of Pest Science* **2017**, *90*, 117-125, doi:10.1007/s10340-016-0769-0.
155. Horgan, F.G.; Romena, A.M.; Bernal, C.C.; Almazan, M.L.P.; Ramal, A.F. Differences between the strength of preference–performance coupling in two rice stemborers (Lepidoptera: Pyralidae, Crambidae) promotes coexistence at field-plot scales. *Environmental Entomology* **2021**, *50*, 929-939. https//doi.org/10.1093/ee/nvab034.
156. Kumar, A.; Ram, L.; Singh, R.; Singh, B. Biology and behavior of white stem borer (S*cirpophaga fusciflua*) on rice (*Oryza sativa*) in India. *Indian Journal of Agricultural Sciences* **2018**, *88*, 129-132.
157. Islam, Z.; Catling, H. Biology and behaviour of rice yellow stem borer in deepwater rice. *Journal of Plant Protection in the Tropics* **1991**, *8*, 85-96.
158. Padmakumari, A.; Katti, G.; Sailaja, V.; Padmavathi, C.; Lakshmi, V.J.; Prabhakar, M.; Prasad, Y. Delineation of larval instars in field populations of rice yellow stem borer, *Scirpophaga incertulas* (Walk.). *ORYZA-An International Journal on Rice* **2013**, *50*, 259-267.
159. Shi, P.J.; Wang, B.; Ayres, M.P.; Ge, F.; Zhong, L.; Li, B.L. Influence of temperature on the northern distribution limits of *Scirpophaga incertulas* Walker (Lepidoptera: Pyralidae) in China. *Journal of Thermal Biology* **2012**, *37*, 130-137, doi:10.1016/j.jtherbio.2011.12.001.
160. Shi, P.J.; Zhong, L.; Sandhu, H.S.; Ge, F.; Xu, X.M.; Chen, W. Population decrease of *Scirpophaga incertulas* Walker (Lepidoptera Pyralidae) under climate warming. *Ecology and Evolution* **2012**, *2*, 58-64. https//doi.org/10.1002/ece3.69.
161. Manikandan, N.; Kennedy, J.S.; Geethalakshmi, V. Effect of elevated temperature on life-history parameters of rice yellow stem borer (*Scirpophaga incertulas* Walker). *Current Science* **2016**, *110*, 851-857.
162. Viswajyothi, K.; Aggarwal, N.; Jindal, J. The biology of Sesamia inferens (Walker)(Lepidoptera: Noctuidae) on maize in the north western plains of India. *Acta Phytopathologica et Entomologica Hungarica* **2019**, *54*, 69-84.
163. Li, C. *The biology and ecology of the white rice stemborer, in northern Australia [Northern Territory]. Technical Bulletin 171.* Northern Territories Department of Primary Industry and Fisheries; Darwin, NT, Australia: 1991.
164. Ruhela, S.K.; Yadav, P.; Kumar, A. Biology of Sugarcane Top Borer, *Scirpophaga nivella* (Lepidoptera). *Annals of Entomology* **2017**, *35*.
